# Supplementary figures and images for: Sylvatic host associations of Triatominae and implications for Chagas disease reservoirs: a review and new host records based on archival specimens
Source: PeerJ. 2017 Sep 18;5:e3826. doi: 10.7717/peerj.3826 (PMC5609523; doi:10.7717/peerj.3826)

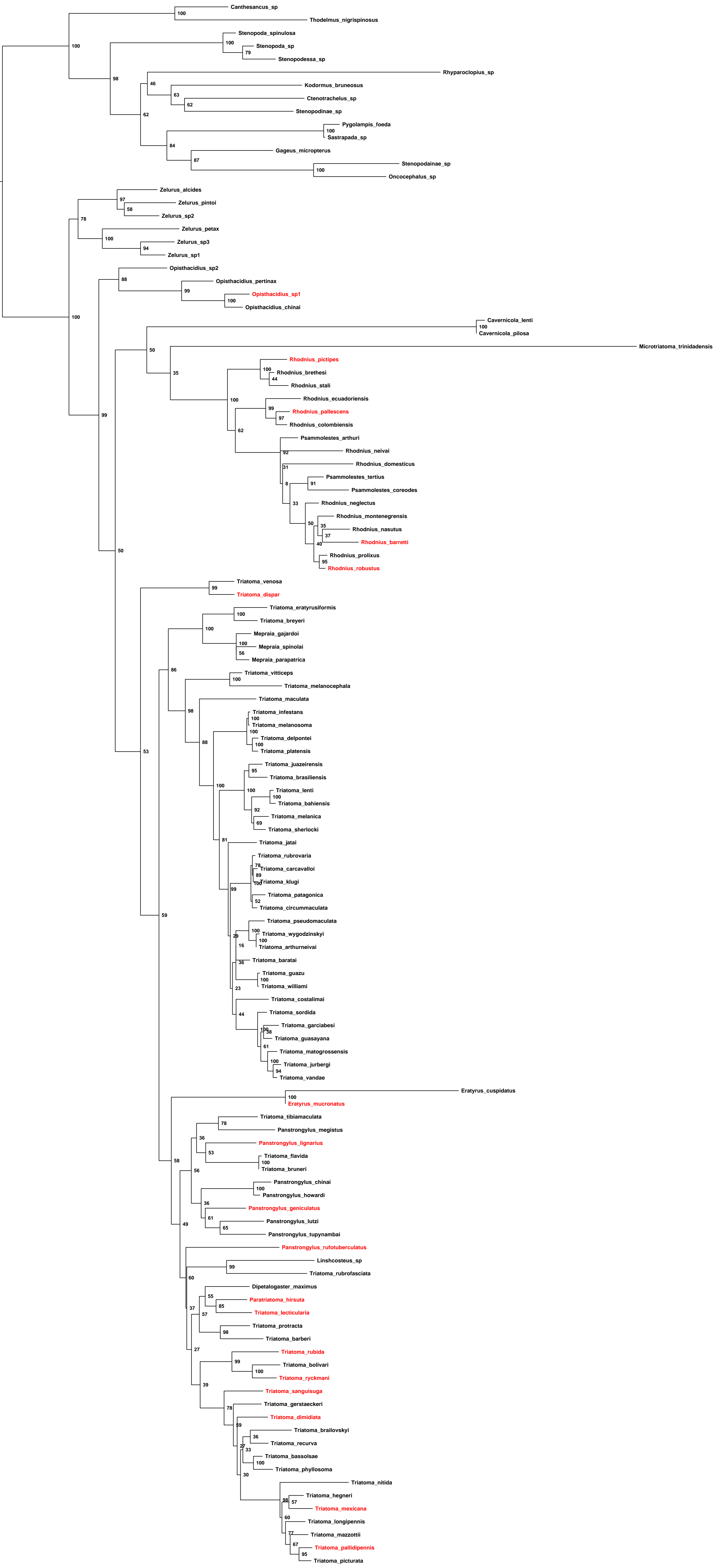

Supplement: Figure S1 — Kissing bug species in red are those for which gut extracts were assayed with primers for vertebrate host and trypanosome DNA. [file peerj-05-3826-s001.pdf]
