# Supplementary material for: Sylvatic host associations of Triatominae and implications for Chagas disease reservoirs: a review and new host records based on archival specimens
Source: PeerJ. 2017 Sep 18;5:e3826. doi: 10.7717/peerj.3826 (PMC5609523; doi:10.7717/peerj.3826)
Supplement: Article S1 [file peerj-05-3826-s003.docx]

Systematic literature review protocol for Triatominae sylvatic hosts references

1. Search the full scientific name of the Triatominae species you are currently working on against the Web of Science “all database” collection under the default “topic” variable. For species sometimes known under both a unique genus name (e.g, *Mepraia,* *Nesotriatoma*) and *Triatoma* search both combinations. Note, that Web of Science “core collection” is the default database and occasionally the database searched may revert to this default, so check each time you search.
2. If the number of results exceeds 500 (*Panstrongylus megistus, Rhonius prolixus, Triatoma brasiliensis, Triatoma dimidiata, Triatoma infestans* and *Triatoma sordida.*), use a more targeted search using the following search terms:

scientific name AND blood meal

scientific name AND host feed*

scientific name AND food

scientific name AND source

scientific name AND gut contents

1. Read the titles of each of the matching results. If the titles include reference to hosts of Triatominae or *Trypanosoma cruzi* or a study or survey of a population of a Triatominae species or a taxonomic work with possible reference to the habitat the insect was collected, click the title and read the abstract.
2. If the abstract indicates that records of any host associations were reported, obtain the full publication via google search of the title or an interlibrary loan request.
3. Read the publication and record any sylvatic host associations found in the paper (excluding domestic animals [cats, dogs, goats, chickens, horses, cows, pigs, sheep] except for rodents including guinea pigs which have been domesticated endemically) and the method with which the association was determined (Associational, Antibody-based or DNA-based) and the reference which the record came from. If the paper cites another paper in reference to a host association, obtain that publication and repeat step 5.
4. Repeat for each search result and each species.

**Notes:**

**Criteria for inclusion:** Records were only excluded if they were very unspecific (e.g., mammals, ungulate, carnivore), if they were the result of a laboratory experiment, if the host was not sylvatic, if the paper surveyed multiple species but did not explicitly state which host associations came from which species, or if later publications explicitly stated that a former association was most likely a mistake. Rarely, host associations originally described for certain populations of one species that were later described as a separate species (e.g., *Mepraia parapatrica*) and we have transferred all relevant host association data. All references to host associations in published papers that met these criteria were included regardless of possible problems with data quality. Studies which assessed the *T. cruzi* infection of various mammals but included no information as to what species of Triatominae were associated with these mammals have not been included because they do not necessarily mean that these animals are hosts of Triatominae as it has been shown that *T. cruzi* can infect animals which eat infected insects or even other *T. cruzi*-infected mammals.

Our first search was conducted from May through July 2016. References and organization were refined over a period of several more months. After our paper was reviewed, we repeated our search in July 2017 to find any more recently published papers.

We did not search for each of the junior synonyms of each species. However, at least in older literature, many of these papers which reference the junior synonym are have been cited by more recent literature under the valid name and can be discovered and read.

While many papers are published in Portuguese (or Spanish), most have a summary or abstract in English that most often allows for assessing whether hosts associations were reported in the paper. If not present in a table, the Portuguese words for common host animals (such as opossums= “gambá” or bird= “pássaro” or “ave”) or other hosts referenced in the abstract were searched for in the text and that section translated using Google translate.

Our institution (University of California, Riverside) subscribes to 14 of the 16 databases provided by Web of Science listed as follows with important databases highlighted: **Web of Science Core Collection**, BIOSIS Citation Index, BIOSIS Previews, **CABI:CAB Abstracts and Global Health**, Chinese Science Citation Database, Current Contents Connect, Data Citation Index, Derwent Innovations Index, Inspec, KCI-Korean Journal Database, **MEDLINE**, Russian Science Citation Index, **SciELO Citation Index, Zoological Record.**

Grey literature such as in Ph.D theses or conference papers may not have been accessible through “Web of Science” searches but we have included references to those that we were made aware of or that were referenced by another published manuscript and for which we could access.
