# Supplementary material for: Sylvatic host associations of Triatominae and implications for Chagas disease reservoirs: a review and new host records based on archival specimens
Source: PeerJ. 2017 Sep 18;5:e3826. doi: 10.7717/peerj.3826 (PMC5609523; doi:10.7717/peerj.3826)
Supplement: Article S2 [file peerj-05-3826-s004.docx]

[Abad-Franch F., Aguilar HM V., Paucar C A., Lorosa ES., Noireau F. 2002. Observations on the domestic ecology of](http://paperpile.com/b/24bkyh/nS7N) *[Rhodnius ecuadoriensis](http://paperpile.com/b/24bkyh/nS7N)* [(Triatominae).](http://paperpile.com/b/24bkyh/nS7N) *[Memórias do Instituto Oswaldo Cruz](http://paperpile.com/b/24bkyh/nS7N)* [97:199–202. DOI:](http://paperpile.com/b/24bkyh/nS7N) [10.1590/S0074-02762002000200010.](http://dx.doi.org/10.1590/S0074-02762002000200010.)

[Abad-Franch F., Monteiro FA., Jaramillo O N., Gurgel-Gonçalves R., Dias FBS., Diotaiuti L. 2009. Ecology, evolution, and the long-term surveillance of vector-borne Chagas disease: a multi-scale appraisal of the tribe Rhodniini (Triatominae). *Acta Tropica* 110:159–177. DOI:](http://paperpile.com/b/24bkyh/bA6O) [10.1016/j.actatropica.2008.06.005.](http://dx.doi.org/10.1016/j.actatropica.2008.06.005.)

[Abalos JW., Wygodzinsky P. 1951. Las Triatominae Argentinas (Reduviidae, Hemiptera). *Publ Inst Med Reg* 601:1–179.](http://paperpile.com/b/24bkyh/rAwz)

[Aguilera X., Miles MA., Apt W. 1986. *Triatoma spinolai* in Chile: a new host for *Hepatozoon triatomae*. *Transactions of the Royal Society of Tropical Medicine and Hygiene* 80:492–493.](http://paperpile.com/b/24bkyh/WSLC)

[Aguirre PE. 1947. Presencia de *Trypanosoma cruzi* en mamíferos y triatomideos de Nuevo Leon, Monterrey, Mexico. *Revista Cuba de Medicina Tropical y Parasitologia* 3:120–121.](http://paperpile.com/b/24bkyh/kl02)

[Alayo P. 1967. Catálogo de la Fauna Cubana. XVI. Los hemípteros de Cuba. III. Familia Reduviidae. *Museo Felipe Poey Academia de Ciencias de Cuba, Trabajos de Divulgación* 41:1–48.](http://paperpile.com/b/24bkyh/S5Vp)

[Aldana E., Viera D., Lizano E. 1997. Morfología de huevos y ninfas de *Psammolestes salazari* (Hemiptera: Reduviidae: Triatominae). *Caribbean J. Sci.* 33:70–74.](http://paperpile.com/b/24bkyh/GvbQ)

[Almeida CE., Duarte R., do Nascimento RG., Pacheco RS., Costa J. 2002. *Triatoma rubrovaria* (Blanchard, 1843) (Hemiptera, Reduviidae, Triatominae) II: trophic resources and ecological observations of five populations collected in the State of Rio Grande do Sul, Brazil.](http://paperpile.com/b/24bkyh/T6Ik) [*Memórias*](http://paperpile.com/b/24bkyh/nS7N) [*do Instituto Oswaldo Cruz* 97:1127–1131.](http://paperpile.com/b/24bkyh/T6Ik)

[Almeida CE., Faucher L., Lavina M., Costa J., Harry M. 2016. Molecular individual-based approach on *Triatoma brasiliensis*: Inferences on triatomine foci, *Trypanosoma cruzi* natural infection prevalence, parasite diversity and feeding sources. *PLoS Neglected Tropical Diseases* 10:e0004447. DOI:](http://paperpile.com/b/24bkyh/Gs0T) [10.1371/journal.pntd.0004447.](http://dx.doi.org/10.1371/journal.pntd.0004447.)

[Alvarado-Otegui JA., Ceballos LA., Orozco MM., Enriquez GF., Cardinal MV., Cura C., Schijman AG., Kitron U., Gürtler RE. 2012. The sylvatic transmission cycle of *Trypanosoma cruzi* in a rural area in the humid Chaco of Argentina. *Acta Tropica* 124:79–86. DOI:](http://paperpile.com/b/24bkyh/jonR) [10.1016/j.actatropica.2012.06.010.](http://dx.doi.org/10.1016/j.actatropica.2012.06.010.)

[de Andrade-Neto OA., de Arruda MCC., Kerkhoff J., Lunardi RR., Arrais-Silva WW. 2012. Risk of domiciliation of *Triatoma williami* Galvão, Souza e Lima, 1965 in a municipality of Brazilian Legal Amazon region. *Asian Pacific Journal of Tropical Disease* 2:S265–S267. DOI:](http://paperpile.com/b/24bkyh/YZ3J) [10.1016/S2222-1808(12)60163-7.](http://dx.doi.org/10.1016/S2222-1808(12)60163-7.)

[Angulo-Silva VM., Castellanos-Domínguez YZ., Flórez-Martínez M., Esteban-Adarme L., Pérez-Mancipe W., Farfán-García AE., Luna-Marín KP. 2016. Human trypanosomiasis in the Eastern Plains of Colombia: New transmission scenario. *The American Journal of Tropical Medicine and Hygiene* 94:348–351. DOI:](http://paperpile.com/b/24bkyh/kd1j) [10.4269/ajtmh.15-0406.](http://dx.doi.org/10.4269/ajtmh.15-0406.)

[Aramburú RM., Berkunsky I., Formoso AE., Cicchino A. 2013. Ectoparasitic load of Blue-crowned Parakeet (*Aratinga a. acuticaudata*, Psittacidae) nestlings. *Ornitología Neotropical* 24:257–265.](http://paperpile.com/b/24bkyh/TjQy)

[Arzube-Rodriguez M. 1966. Investigación de la fuente alimenticia del *T. dimidiata*, Latr. 1811 (Hemiptera: Reduviidae), mediante la reacción de precipitina. *Revista Ecuatoriana de Higiene y Medicina Tropical* 23:137–152.](http://paperpile.com/b/24bkyh/W885)

[Avendano-Rangel F., Pefaur J., Lizano E., Aldana E., Velasquez-Olivares D., Concepción JL. 2011. *Eratyrus mucronatus* (Hemiptera, Triatominae) domiciliated and fed with human and dog blood in Merida state, Venezuela: A potential risk in the transmission of the Chagas disease: Technical note. *Revista Científica de Veterinaria* 21:421–424.](http://paperpile.com/b/24bkyh/aluX)

[Barbosa-Silva AN., Câmara ACJ da., Martins K., Nunes DF., Oliveira PIC de., Azevedo PRM de., Chiari E., Galvão LM da C. 2016. Characteristics of Triatomine infestation and natural *Trypanosoma cruzi* infection in the State of Rio Grande do Norte, Brazil. *Revista da Sociedade Brasileira de Medicina Tropical* 49:57–67. DOI:](http://paperpile.com/b/24bkyh/up9p) [10.1590/0037-8682-0300-2015.](http://dx.doi.org/10.1590/0037-8682-0300-2015.)

[Barrett TV. 1991. Advances in triatomine bug ecology in relation to Chagas’ disease. In: *Advances in disease vector research*. 143–176.](http://paperpile.com/b/24bkyh/xGDG)

[Barrett TV., Hoff RH., Mott KE., Miles MA., Godfrey DG., Teixeira R., Almeida de Souza JA., Sherlock IA. 1980. Epidemiological aspects of three *Trypanosoma cruzi* zymodemes in Bahia State, Brazil. *Transactions of the Royal Society of Tropical Medicine and Hygiene* 74:84–90.](http://paperpile.com/b/24bkyh/imEH)

[Barretto MP. 1967. Estudos sôbre reservatórios e vectores silvestres do *Trypanosoma cruzi*. XVII. Contribuição para o estudo dos focos naturais da tripanossomose americana, com especial referência à região nordeste do Estado de São Paulo, Brasil. *Revista da Sociedade Brasileira de Medicina Tropical* 1:23–35.](http://paperpile.com/b/24bkyh/rrVx)

[Barretto MP. 1968. Estudos sôbre reservatórios e vectores silvestres do *Trypanosoma cruzi*. XXXI. Observações sobre a associação entre reservatórios e vectores, com especial referência à região nordeste do estado de São Paulo. *Revista Brasileira de Biologia* 28:481–494.](http://paperpile.com/b/24bkyh/zZuK)

[Barretto MP. 1971. Estudos sôbre reservatórios e vectores silvestres do *Trypanosoma cruzi*. XLV. Inquérito preliminar sobre triatomíneos silvestres no sul do Estado de Mato Grosso, Brasil (Hemiptera, Reduviidae). *Revista Brasileira de Biologia* 31:225–233.](http://paperpile.com/b/24bkyh/U6Fq)

[Barretto MP., Carvalheiro JR. 1966. Estudos sôbre reservatórios e vectores silvestres do *Trypanosoma cruzi*. XII. Inquérito preliminar sôbre triatomíneos silvestres no Municipio de Uberaba, Minas Gerais. *Revista Brasileira de Biologia* 26:5–14.](http://paperpile.com/b/24bkyh/88ZZ)

[Barretto MP., Carvalheiro JR. 1967. Estudos sôbre reservatórios e vectores silvestres do *Trypanosoma cruzi*. XVIII. Observações sobre a ecologia do *Psammolestes tertius* Lent & Jurberg, 1965 (Hemiptera, Reduviidae). *Revista Brasileira de Biologia* 27:13–25.](http://paperpile.com/b/24bkyh/WIIN)

[Barretto MP., Carvalheiro JR. 1968. Estudos sobre reservatórios e vectores silvestres do *Trypanosoma cruzi*. XXVIII: sobre o encontro de *Triatoma sordida* Stal, 1859 e de *Rhodnius neglectus* Lent, 1954 em ninhos de pássaros da família Furnariidae (Hemiptera, Reduviidae). *Revista Brasileira de Biologia* 28:289–293.](http://paperpile.com/b/24bkyh/41mt)

[Barretto MP., Ribeiro RD. 1981. Estudos sobre reservatorios e vectores silvestres do *Trypanosoma cruzi*. LXXVII: Observações sobre a ecologia do *Triatoma arthurneivai* Lent & Martins, 1940. *Revista Brasileira de Biologia* 41:317–320.](http://paperpile.com/b/24bkyh/SVNh)

[Barretto MP., Siqueira AF., Ferriolli Filho F., Carvalheiro JR. 1966. Estudos sobre reservatórios e vectores silvestres do *Trypanosoma cruzi.* XI. Observações sobre um foco natural da tripanossomose americana no município de Ribeirão Preto, São Paulo. *Revista do Instituto de Medicina Tropical de Sao Paulo* 8:103–112.](http://paperpile.com/b/24bkyh/Nkx6)

[Barros GC., Mayrink W., Salgado AA., Barros R., Sessa PA. 1975. Contribuição para o conhecimento da doença de Chagas autóctone no estado do Espírito Santo. *Revista do Instituto de Medicina Tropical de Sao Paulo* 17:319–329.](http://paperpile.com/b/24bkyh/zQiL)

[Bar ME., Wisnivesky-Colli C. 2001. *Triatoma sordida* Stål 1859 (Hemiptera, Reduviidae: Triatominae) in palms of northeastern Argentina. *Memórias do Instituto Oswaldo Cruz* 96:895–899. DOI:](http://paperpile.com/b/24bkyh/G3ew) [10.1590/S0074-02762001000700002.](http://dx.doi.org/10.1590/S0074-02762001000700002.)

[Bento DN da C., Branco AZCL., Freitas MR., Pinto A da S. 1984. Epidemiologic studies of Chagas disease in the urban zone of Teresina. State of Piauí, northeastern Brazil. *Revista da Sociedade Brasileira de Medicina Tropical* 17:199–203. DOI:](http://paperpile.com/b/24bkyh/xepB) [10.1590/S0037-86821984000400006.](http://dx.doi.org/10.1590/S0037-86821984000400006.)

[Bezerra CM., Cavalcanti LP de G., Souza R de CM de., Barbosa SE., Xavier SC das C., Jansen AM., Ramalho RD., Diotaiut L. 2014. Domestic, peridomestic and wild hosts in the transmission of *Trypanosoma cruzi* in the Caatinga area colonised by *Triatoma brasiliensis*. *Memórias do Instituto Oswaldo Cruz* 109:887–898.](http://paperpile.com/b/24bkyh/DDB1)

[Biagi F., Tay J., Guzman-Garcia C., Fong P. 1964. Tetitlan, Guerrero, foco endemico de enfermedad de Chagas en Mexico. *Revista de la Facultad de Medicina, Universidad Nacional Autonoma de Mexico* 6:625–631.](http://paperpile.com/b/24bkyh/x08u)

[Bonne C. 1937. The Natural Host of Trypanosoma (Crithidia) Conorhini Donovan. *The American Journal of Tropical Medicine and Hygiene* s1-17:393–399. DOI:](http://paperpile.com/b/24bkyh/LfEI) [10.4269/ajtmh.1937.s1-17.393.](http://dx.doi.org/10.4269/ajtmh.1937.s1-17.393.)

[Bosseno M-F., Barnabé C., Sierra MJR., Kengne P., Guerrero S., Lozano F., Ezequiel K., Gastélum M., Brenière SF. 2009. Wild ecotopes and food habits of *Triatoma longipennis* infected by *Trypanosoma cruzi* lineages I and II in Mexico. *The American Journal of Tropical Medicine and Hygiene* 80:988–991.](http://paperpile.com/b/24bkyh/fP1C)

[Bosseno M-F., García LS., Baunaure F., Gastelúm EM., Gutierrez MS., Kasten FL., Dumonteil E., Brenière SF. 2006. Identification in triatomine vectors of feeding sources and *Trypanosoma cruzi* variants by heteroduplex assay and a multiplex miniexon polymerase chain reaction. *The American Journal of Tropical Medicine and Hygiene* 74:303–305.](http://paperpile.com/b/24bkyh/Gojp)

[Botto-Mahan C., Cattan PE., Canals M., Acuña M. 2005. Seasonal variation in the home range and host availability of the blood-sucking insect *Mepraia spinolai* in wild environment. *Acta Tropica* 95:160–163. DOI:](http://paperpile.com/b/24bkyh/Dcdj) [10.1016/j.actatropica.2005.05.001.](http://dx.doi.org/10.1016/j.actatropica.2005.05.001.)

[Botto-Mahan C., Sepúlveda M., Vidal M., Acuña-Retamar M., Ortiz S., Solari A. 2008. *Trypanosoma cruzi* infection in the sylvatic kissing bug *Mepraia gajardoi* from the Chilean Southern Pacific Ocean coast. *Acta Tropica* 105:166–169. DOI:](http://paperpile.com/b/24bkyh/mCWZ) [10.1016/j.actatropica.2007.11.003.](http://dx.doi.org/10.1016/j.actatropica.2007.11.003.)

[Brenière SF., Pietrokovsky S., Gastélum EM., Bosseno M-F., Soto MM., Ouaissi A., Kasten FL., Wisnivesky-Colli C. 2004. Feeding patterns of *Triatoma longipennis* Usinger (Hemiptera, Reduviidae) in peridomestic habitats of a rural community in Jalisco State, Mexico. *Journal of Medical Entomology* 41:1015–1020.](http://paperpile.com/b/24bkyh/eoZU)

[Brewer M., Garay M., Gorla D., Murua F., Favot R. 1981. Caracterización de los estadíos ninfales del género *Triatoma* (Laporte, 1833). I. *Triatoma infestans* Klug, 1834 (Hemiptera: Reduviidae). *Revista de la Sociedad Entomologica Argentina* 40:91–102.](http://paperpile.com/b/24bkyh/vT9i)

[Brumpt E., Mazzoti L., Brumpt LC. 1939. Enquêtes épidémiologiques sur la maladie de C. Chagas au Mexique. Reduvides vecteurs, animaux réservoirs de virus, cas humains. *Annals of parasitology* 17:299–312.](http://paperpile.com/b/24bkyh/70Ur)

[Bruner SC. 1926. Synopsis of Cuban Reduviidae. *Memorias de la Sociedad Cubana de Historia Natural “Felipe Poey.”* 7:65–82.](http://paperpile.com/b/24bkyh/Evc0)

[Buitrago R., Bosseno MF., Depickère S., Waleckx E., Salas R., Aliaga C., Barnabé C., Brenière SF. 2016. Blood meal sources of wild and domestic *Triatoma infestans* (Hemiptera: Reduviidae) in Bolivia: connectivity between cycles of transmission of *Trypanosoma cruzi*. *Parasites & vectors* 9:214. DOI:](http://paperpile.com/b/24bkyh/gDo4) [10.1186/s13071-016-1499-0.](http://dx.doi.org/10.1186/s13071-016-1499-0.)

[Buitrago NLR., Bosseno MF., Waleckx E., Brémond P., Vidaurre P., Zoveda F., Brenière SF. 2013. Risk of transmission of Trypanosoma cruzi by wild *Triatoma infestans* (Hemiptera: Reduviidae) in Bolivia supported by the detection of human blood meals. *Infection, Genetics and Evolution: Journal of Molecular Epidemiology and Evolutionary Genetics in Infectious Diseases* 19:141–144. DOI:](http://paperpile.com/b/24bkyh/p7PQ) [10.1016/j.meegid.2013.07.002.](http://dx.doi.org/10.1016/j.meegid.2013.07.002.)

[Bustamante DM., De Urioste-Stone SM., Juárez JG., Pennington PM. 2014. Ecological, social and biological risk factors for continued *Trypanosoma cruzi* transmission by *Triatoma dimidiata* in Guatemala. *PloS One* 9:e104599. DOI:](http://paperpile.com/b/24bkyh/KswO) [10.1371/journal.pone.0104599.](http://dx.doi.org/10.1371/journal.pone.0104599.)

[Cáceres AG., Vega S., Ancca J., Pinto J., Vela G., Cárdenas V., Ruiz J., Del Pilar Alva P., Ruiz J., Alvarado A., Arévalo H., Cruzado F., Vela F., Náquira C. 2010. Aspectos entomológicos de la enfermedad de Chagas en Huallaga y Picota, San Martín, Perú. *Anales de la Facultad de Medicina* 71:28–36. DOI:](http://paperpile.com/b/24bkyh/EN1M) [10.15381/anales.v71i1.69.](http://dx.doi.org/10.15381/anales.v71i1.69.)

[Calderón-Arguedas O., Chinchílla M., García F., Vargas M. 2001. Preferencias alimentarias de *Triatoma dimidiata* (Hemiptera: Reduvíidae) procedente de la meseta central de Costa Rica a finales del siglo XX. *Parasitología al día* 25:78–81. DOI:](http://paperpile.com/b/24bkyh/ixQr) [10.4067/S0716-07202001000300002.](http://dx.doi.org/10.4067/S0716-07202001000300002.)

[Calegari L., Salvatella R., Guerrero J., Puime A., Basmadjian Y., Rosa R. 1995. Hábitos alimentarios de *Triatoma rubrovaria* (Blanchard, 1843) (Hemiptera, Triatominae), en diferentes situaciones ecoepidemiológicas. *Boletín de la Sociedad Zoológica del Uruguay* 9:61–66.](http://paperpile.com/b/24bkyh/8xY8)

[Canals M., Cruzat L., Molina MC., Ferreira A., Cattan PE. 2001. Blood host sources of *Mepraia spinolai* (Heteroptera: Reduviidae), wild vector of chagas disease in Chile. *Journal of Medical Entomology* 38:303–307. DOI:](http://paperpile.com/b/24bkyh/brXM) [10.1603/0022-2585-38.2.303.](http://dx.doi.org/10.1603/0022-2585-38.2.303.)

[Cantillo-Barraza O., Garcés E., Gómez-Palacio A., Cortés LA., Pereira A., Marcet PL., Jansen AM., Triana-Chávez O. 2015. Eco-epidemiological study of an endemic Chagas disease region in northern Colombia reveals the importance of *Triatoma maculata* (Hemiptera: Reduviidae), dogs and *Didelphis marsupialis* in *Trypanosoma cruzi* maintenance. *Parasites & vectors* 8:482. DOI:](http://paperpile.com/b/24bkyh/az4i) [10.1186/s13071-015-1100-2.](http://dx.doi.org/10.1186/s13071-015-1100-2.)

[Caranha L., Lorosa ES., Rocha D da S., Jurberg J., Galvão C. 2006. Feeding sources evaluation of *Panstrongylus lutzi* (Neiva & Pinto, 1923) (Hemiptera: Reduviidae: Triatominae) in the State of Ceará. *Revista da Sociedade Brasileira de Medicina Tropical* 39:347–351. DOI:](http://paperpile.com/b/24bkyh/qpVw) [10.1590/S0037-86822006000400006.](http://dx.doi.org/10.1590/S0037-86822006000400006.)

[Carcavallo RU. 1987. The subfamily Triatominae (Hemiptera, Reduviidae): systematics and some ecological factors. In: Brenner RR, de la Merced Stoka A eds. *Chagas’ disease vectors. Volume 1. Taxonomic, Ecological and Epidemiological Aspects.* 1–20.](http://paperpile.com/b/24bkyh/mLbl)

[Carcavallo RU., Barata JMS., Costa AIP da., Serra OP. 1995. *Alberprosenia malheiroi* Serra, Atzingen & Serra, 1987 (Hemiptera, Reduviidae): redescription and bionomics. *Revista de Saúde Pública* 29:488–495. DOI:](http://paperpile.com/b/24bkyh/OBfu) [10.1590/S0034-89101995000600010.](http://dx.doi.org/10.1590/S0034-89101995000600010.)

[Carcavallo RU., Barreto P., Martinez A., Tonn R. 1976. El genero *Microtriatoma* Prosen y Martinez, 1952 (Hemiptera, Reduviidae). *Bol Dir Malariol Saneam* 16:231–240.](http://paperpile.com/b/24bkyh/lCWN)

[Carcavallo RU., Martínez A. 1985. Biología, ecología y distribución geográfica de los triatominos americanos. In: Carcavallo RU, Rabinovich JE, Tonn RJ eds. *Factores Biológicos y Ecológicos en la Enfermedad de Chagas, Vol. I*. Ministerio de Salud y Acción Social de Argentina, Buenos Aires, 149–208.](http://paperpile.com/b/24bkyh/a3SA)

[Carcavallo R., Otero MA., Tonn RJ., Ortega R. 1975. Notas sobre la biología, ecología y distribución geográfica del *Psammolestes arthuri* (Pinto), 1926 (Hemiptera, Reduviidae). Descripción de los estadios imaginales. *Boletín Informativo de la Dirección de Malariología y Saneamiento Ambiental* 15:231–239.](http://paperpile.com/b/24bkyh/hd1P)

[Carcavallo RU., da Silva Rocha D., Galindez Giron I. 1998. Feeding sources and patterns. In: Carcavallo RU, Galindez Giron I, Jurberg J eds. *Atlas of Chagas’ disease vectors in the Americas. Vol. 2.* 537–560.](http://paperpile.com/b/24bkyh/KCQz)

[Carcavallo RU., Tonn R. 1985. Capítulo XV. *Rhodnius prolixus*. In: Carcavallo RU, Rabinovich JE, Tonn RJ eds. *Factores biológicos y ecológicos en la enfermedad de Chagas*. Tomo I - Epidemiología - Vectores,. 209–217.](http://paperpile.com/b/24bkyh/Fd4a)

[Cardoso SFM. 2006. Avaliação de vetores da Doença de Chagas na área de influência direta da usina hidrelétrica Corumbá IV, Goiás. Universidade Católica de Goiás. Goiânia, Goiás.](http://paperpile.com/b/24bkyh/hWkJ)

[Carrasco HJ., Torrellas A., García C., Segovia M., Feliciangeli MD. 2005. Risk of *Trypanosoma cruzi* I (Kinetoplastida: Trypanosomatidae) transmission by *Panstrongylus geniculatus* (Hemiptera: Reduviidae) in Caracas (Metropolitan District) and neighboring States, Venezuela. *International Journal for Parasitology* 35:1379–1384. DOI:](http://paperpile.com/b/24bkyh/ESZd) [10.1016/j.ijpara.2005.05.003.](http://dx.doi.org/10.1016/j.ijpara.2005.05.003.)

[Castro-Ferreira L., Deane L. 1938. Encontro de um novo hematófago do homem com hábitos domiciliares. *Brasil médico* 52:1137–1141.](http://paperpile.com/b/24bkyh/gzpA)

[Ceballos LA., Piccinali RV., Berkunsky I., Kitron U., Gürtler RE. 2009. First finding of melanic sylvatic *Triatoma infestans* (Hemiptera: Reduviidae) colonies in the Argentine Chaco. *Journal of Medical Entomology* 46:1195–1202.](http://paperpile.com/b/24bkyh/lR5e)

[Cecere MC., Leporace M., Fernández MP., Zárate JE., Moreno C., Gürtler RE., Cardinal MV. 2016. Host-Feeding Sources and Infection With *Trypanosoma cruzi* of *Triatoma infestans* and *Triatoma eratyrusiformis* (Hemiptera: Reduviidae) From the Calchaqui Valleys in Northwestern Argentina. *Journal of Medical Entomology*. DOI:](http://paperpile.com/b/24bkyh/OSnW) [10.1093/jme/tjw002.](http://dx.doi.org/10.1093/jme/tjw002.)

[Chacón F., Bacigalupo A., Quiroga JF., Ferreira A., Cattan PE., Ramírez-Toloza G. 2016. Feeding profile of *Mepraia spinolai*, a sylvatic vector of Chagas disease in Chile. *Acta Tropica* 162:171–173. DOI:](http://paperpile.com/b/24bkyh/f44F) [10.1016/j.actatropica.2016.06.027.](http://dx.doi.org/10.1016/j.actatropica.2016.06.027.)

[Christensen HA., Sousa OE., de Vasquez AM. 1988. Host feeding profiles of *Triatoma dimidiata* in peridomestic habitats of western Panama. *The American Journal of Tropical Medicine and Hygiene* 38:477–479.](http://paperpile.com/b/24bkyh/zRhK)

[Christensen HA., de Vasquez AM. 1981. Host feeding profiles of *Rhodnius pallescens* (Hemiptera: Reduviidae) in rural villages of Central Panama. *The American Journal of Tropical Medicine and Hygiene* 30:278–283.](http://paperpile.com/b/24bkyh/byqW)

[Christensen HA., Whitlaw JT., Chaniotis BN., De VÁSquez AM. 1980. Sylvatic hosts of *Rhodnius pallescens* (Hemiptera: Reduviidae) nymphs in the Panama canal zone. *Journal of Medical Entomology* 17:182–182. DOI:](http://paperpile.com/b/24bkyh/3N25) [10.1093/jmedent/17.2.182.](http://dx.doi.org/10.1093/jmedent/17.2.182.)

[Cichero JA., Carcavallo RU. 1967. Notas sobre la bioecología del *Triatoma breyeri* Del Ponte, 1929. *Neotropica* 13:52–53.](http://paperpile.com/b/24bkyh/Xcpc)

[Correa RR., Aguiar AA. 1952. O teste de precipitina na identificação da fonte alimentar do *Triatoma infestans* (Hemiptera, Reduviidae). *Arquivos de higiene e saúde pública* 17:3–8.](http://paperpile.com/b/24bkyh/VE5k)

[Costa J., de Almeida JR., Britto C., Duarte R., Marchon-Silva V., Pacheco R da S. 1998. Ecotopes, natural infection and trophic resources of *Triatoma brasiliensis*  (Hemiptera, Reduviidae, Triatominae). *Memórias do Instituto Oswaldo Cruz* 93:7–13. DOI:](http://paperpile.com/b/24bkyh/oZY0) [10.1590/S0074-02761998000100002.](http://dx.doi.org/10.1590/S0074-02761998000100002.)

[Curtis-Robles R., Lewis BC., Hamer SA. 2016. High *Trypanosoma cruzi* infection prevalence associated with minimal cardiac pathology among wild carnivores in central Texas. *International journal for parasitology: Parasites and wildlife* 5:117–123. DOI:](http://paperpile.com/b/24bkyh/kqNg) [10.1016/j.ijppaw.2016.04.001.](http://dx.doi.org/10.1016/j.ijppaw.2016.04.001.)

[D’Alessandro A., Barreto P., Duarte CA. 1971. Distribution of triatomine-transmitted trypanosomiasis in Colombia and new records of the bugs and infections. *Journal of Medical Entomology* 8:159–172. DOI:](http://paperpile.com/b/24bkyh/1ccp) [10.1093/jmedent/8.2.159.](http://dx.doi.org/10.1093/jmedent/8.2.159.)

[D’Alessandro A., Barreto P., Thomas M. 1981. Nuevos registros de triatominos domiciliarios y extradomiciliarios en Colombia. *Colombia médica* 12:75–85.](http://paperpile.com/b/24bkyh/bFzB)

[De Almeida PS., Dos Santos HR., Barata JMS., Obara MT., Ceretti W. 2008. Occurrence of *Panstrongylus guentheri* Berg. (Hemiptera: Reduviidae) in Mato Grosso do Sul State, Brazil. *Neotropical entomology* 37:107–108.](http://paperpile.com/b/24bkyh/r2Ui)

[De la Riva J., Matias A., Torrez M., Martínez E., Dujardin JP. 2001. Adult and nymphs of *Microtriatoma trinidadensis* (Lent, 1951) (Hemiptera: Reduviidae) caught from peridomestic environment in Bolivia. *Memórias do Instituto Oswaldo Cruz* 96:889–894. DOI:](http://paperpile.com/b/24bkyh/8CRj) [10.1590/S0074-02762001000700001.](http://dx.doi.org/10.1590/S0074-02762001000700001.)

[Dias FBS., Bezerra CM., Machado EM de M., Casanova C., Diotaiuti L. 2008. Ecological aspects of *Rhodnius nasutus* Stål, 1859 (Hemiptera: Reduviidae: Triatominae) in palms of the Chapada do Araripe in Ceará, Brazil. *Memórias do Instituto Oswaldo Cruz* 103:824–830. DOI:](http://paperpile.com/b/24bkyh/AJYS) [10.1590/S0074-02762008000800014.](http://dx.doi.org/10.1590/S0074-02762008000800014.)

[Dias-Lima AG., Menezes D., Sherlock I., Noireau F. 2003. Wild habitat and related fauna of *Panstrongylus lutzi* (Reduviidae, Triatominae). *Journal of Medical Entomology* 40:989–990. DOI:](http://paperpile.com/b/24bkyh/lJXl) [10.1603/0022-2585-40.6.989.](http://dx.doi.org/10.1603/0022-2585-40.6.989.)

[Dias E., Mello GB., Costa D., Damasceno R., Azevedo M. 1942. Investigações sobre esquisotripanose de morcegos no Estado do Pará. Encontro do barbeiro *Cavernicola pilosa* como transmissor. *Revista Brasileira de Biologia* 2:103–110.](http://paperpile.com/b/24bkyh/k30q)

[Dias FBS., Quartier M., Romaña CA., Diotaiuti L., Harry M. 2010. *Tamandua tetradactyla* Linnaeus, 1758 (Myrmecophagidae) and *Rhodnius robustus* Larrousse, 1927 (Triatominae) infection focus by *Trypanosoma rangeli* Tejera, 1920 (Trypanosomatidae) in *Attalea phalerata* Mart. ex Spreng (Arecaceae) palm tree in the Brazilian Amazon. *Infection, genetics and evolution* 10:1278–1281. DOI:](http://paperpile.com/b/24bkyh/UqSF) [10.1016/j.meegid.2010.06.020.](http://dx.doi.org/10.1016/j.meegid.2010.06.020.)

[Díaz Cruz SJ. 2017. Caracterización de hábitos alimentarios en *Triatoma dimidiata* mediante la técnica de PCR forense en el municipio de Texistepeque, departamento de Santa Ana. Bachelor Thesis Thesis. Universidad de El Salvador. Available at http://ri.ues.edu.sv/13951/1/19201064.pdf (accessed 9 August 2017).](http://paperpile.com/b/24bkyh/GyYJ)

[Diotaiuti L., Loiola CF., Falcão PL., Dias JCP. 1993. The ecology of *Triatoma sordida* in natural environments in two different regions of the state of Minas Gerais, Brazil. *Revista do Instituto de Medicina Tropical de Sao Paulo* 35:237–245. DOI:](http://paperpile.com/b/24bkyh/AIpv) [10.1590/S0036-46651993000300004.](http://dx.doi.org/10.1590/S0036-46651993000300004.)

[Di Primio R. 1966. Presence of *Triatoma circummaculata* in Porto Alegre-other observations. *Anais da Faculdade de Medicina de Porto Alegre* 26:73–77.](http://paperpile.com/b/24bkyh/LTEk)

[Dolhun EP., Antes AW. 2016. A Case of Cardboard Boxes Likely Facilitating the Biting of a Patient by *Trypanosoma cruzi*-Infected Triatomine Bugs. *The American Journal of Tropical Medicine and Hygiene* 95:1115–1117. DOI:](http://paperpile.com/b/24bkyh/yGv2) [10.4269/ajtmh.16-0455.](http://dx.doi.org/10.4269/ajtmh.16-0455.)

[Durán P., Siñani E., Depickère S. 2016. On triatomines, cockroaches and haemolymphagy under laboratory conditions: new discoveries. *Memórias do Instituto Oswaldo Cruz* 111:605–613. DOI:](http://paperpile.com/b/24bkyh/SOFV) [10.1590/0074-02760160027.](http://dx.doi.org/10.1590/0074-02760160027.)

[Elkins JC. 1951. The Reduviidae of Texas. *The Texas journal of science* 4:307–312.](http://paperpile.com/b/24bkyh/K2nM)

[Fairchild GB. 1943. An annotated list of the bloodsucking insects, ticks, and mites known from Panama. *The American Journal of Tropical Medicine and Hygiene* 23:569–591.](http://paperpile.com/b/24bkyh/x0eC)

[Farfán AE., Gutiérrez R., Angulo VM. 2007. Using ELISA for identifying Triatominae feeding behaviour in Colombia. *Revista de Salud Pública* 9:602–608. DOI:](http://paperpile.com/b/24bkyh/7RDD) [10.1590/S0124-00642007000400013.](http://dx.doi.org/10.1590/S0124-00642007000400013.)

[Feliciangeli MD., Dujardin J-P., Bastrenta B., Mazzarri M., Villegas J., Flores M., Muñoz M. 2002. Is *Rhodnius robustus* (Hemiptera: Reduviidae) responsible for Chagas disease transmission in Western Venezuela? *Tropical medicine & international health: TM & IH* 7:280–287.](http://paperpile.com/b/24bkyh/I9bt)

[Floch H., Abonnenc E. 1942. Trypanosomes evoluant dans le tube digestif do *R. prolixus*. *Publications De L’institute Pasteur De La Guyane* 54:1–2.](http://paperpile.com/b/24bkyh/Bcq5)

[Forattini OP., Barata JM., Santos JL., Silveira AC. 1981. Hábitos alimentares, infecção natural e distribuição de triatomíneos domiciliados na região nordeste do Brasil. *Revista de saude publica* 15:113–164.](http://paperpile.com/b/24bkyh/mRVR)

[Forattini OP., Barata JMS., Santos JLF., Silveira AC. 1982. Hábitos alimentares, infecção natural e distribuição de triatomíneos domiciliados na região central do Brasil. *Revista de Saúde Pública* 16:171–204.](http://paperpile.com/b/24bkyh/8DDQ)

[Forattini OP., Juarez E., Rabello EX. 1968. Dados sôbre a biologia do *Triatoma arthurneivai* no sudeste do Estado de São Paulo, Brasil (Hemiptera, Reduviidae). *Revista de Saúde Pública* 2:186–193. DOI:](http://paperpile.com/b/24bkyh/rM4x) [10.1590/S0034-89101968000200005.](http://dx.doi.org/10.1590/S0034-89101968000200005.)

[Forattini OP., Rocha e Silva EO da., Ferreira OA., Rabello EX., Pattoli DGB. 1971. Ecological aspects of South American trypanosomiasis: III - local dispersion of triatomids, with special reference to *Triatoma sordida*. *Revista de Saúde Pública* 5:193–205. DOI:](http://paperpile.com/b/24bkyh/3D4v) [10.1590/S0034-89101971000200002.](http://dx.doi.org/10.1590/S0034-89101971000200002.)

[Freitas SPC., Lorosa ES., Rodrigues DCS., Freitas ALC., Gonçalves TCM. 2005. Feeding patterns of *Triatoma pseudomaculata* in the state of Ceará, Brazil. *Revista de Saúde Pública* 39:27–32. DOI:](http://paperpile.com/b/24bkyh/thu5) [10.1590/S0034-89102005000100004.](http://dx.doi.org/10.1590/S0034-89102005000100004.)

[Freitas J., Siqueira AF., Ferreira OA. 1960. Investigações epidemiológicas sobre triatomíneos de hábitos domésticos e silvestres com auxílio da reação de precipitina. *Revista do Instituto de Medicina Tropical de Sao Paulo* 2:90–99.](http://paperpile.com/b/24bkyh/SqYk)

[Frías-Lasserre D. 2010. A new species and karyotype variation in the bordering distribution of *Mepraia spinolai* (Porter) and *Mepraia gajardoi* Frías et al (Hemiptera: Reduviidae: Triatominae) in Chile and its parapatric model of speciation. *Neotropical entomology* 39:572–583.](http://paperpile.com/b/24bkyh/l7Xa)

[Galindo P., Fairchild GB. 1962. Notes on habits of two bloodsucking bugs, *Triatoma dispar* Lent, 1950, and *Eratyrus cuspidatus* Stal, 1859 (Hemiptera: Reduviidae). *Proceedings of the Entomological Society of Washington* 64:229–230.](http://paperpile.com/b/24bkyh/XItc)

[Galloway CB. 1973. *Forty-fourth annual report of the work and operations of the Gorgas Memorial Laboratory, fiscal year 1972*. U.S. Government Printing Office.](http://paperpile.com/b/24bkyh/PGBB)

[Gamboa CI. 1965. Comprobación de *Rhodnius prolixus* extradoméstico en Venezuela. *Archivos venezolanos de medicina tropical y parasitología médica* 5:283–298.](http://paperpile.com/b/24bkyh/J1sX)

[Gamboa CJ. 1970. La población silvestre de *Rhodnius prolixus* en Venezuela. *Boletin Informativo de la Direccion de Malariologia y Saneamiento Ambiental* 10:186–207.](http://paperpile.com/b/24bkyh/MPOm)

[Garcia MHM., Pinto CT., Lorosa ES., Souza R de CM de., Diotaiuti L. 2013. Spraying food sources with pyrethroid to control peridomestic triatomines. *Revista da Sociedade Brasileira de Medicina Tropical* 46:633–636. DOI:](http://paperpile.com/b/24bkyh/5cSc) [10.1590/0037-8682-1614-2013.](http://dx.doi.org/10.1590/0037-8682-1614-2013.)

[Garrouste R. 2009. La première observation in natura de l’entomophagie de Panstrongylus geniculatus (Latreille 1811) hématophage vecteur de la maladie de Chagas (Hemiptera: Reduviidae). *Annales de la Societe entomologique de France. Societe entomologique de France* 45:302–304. DOI:](http://paperpile.com/b/24bkyh/9hib) [10.1080/00379271.2009.10697614.](http://dx.doi.org/10.1080/00379271.2009.10697614.)

[Gaunt M., Miles M. 2000. The ecotopes and evolution of triatomine bugs (Triatominae) and their associated trypanosomes. *Memórias do Instituto Oswaldo Cruz* 95:557–565. DOI:](http://paperpile.com/b/24bkyh/4T1f) [10.1590/S0074-02762000000400019.](http://dx.doi.org/10.1590/S0074-02762000000400019.)

[Gilbert C., Schaack S., Pace JK 2nd., Brindley PJ., Feschotte C. 2010. A role for host-parasite interactions in the horizontal transfer of transposons across phyla. *Nature* 464:1347–1350. DOI:](http://paperpile.com/b/24bkyh/laGP) [10.1038/nature08939.](http://dx.doi.org/10.1038/nature08939.)

[Gonçalves TC., Rocha DS., Cunha RA. 2000. Feeding patterns of *Triatoma vitticeps* in the State of Rio de Janeiro, Brazil. *Revista de saude publica* 34:348–352. DOI:](http://paperpile.com/b/24bkyh/b1v4) [10.1590/S0034-89102000000400006.](http://dx.doi.org/10.1590/S0034-89102000000400006.)

[Gonzalez-Angulo W., Ryckman RE. 1967. Epizootiology of *Trypanosoma cruzi* in southwestern North America. IX. An investigation to determine the incidence of *Trypanosoma cruzi* infections in Triatominae and man on the Yucatan Peninsula of Mexico. *Journal of Medical Entomology* 4:44–47.](http://paperpile.com/b/24bkyh/x4NH)

[González-Britez N., Vega C., Rolón M., Rojas de Arias A. 2000. Triatomines and other arthropods in bird nests in native and Mennonite communities of the paraguayan Chaco. 2:89.](http://paperpile.com/b/24bkyh/Lru2)

[Gorchakov R., Trosclair LP., Wozniak EJ., Feria PT., Garcia MN., Gunter SM., Murray KO. 2016. *Trypanosoma cruzi* infection prevalence and bloodmeal analysis in triatomine vectors of Chagas disease from rural peridomestic locations in Texas, 2013–2014. *Journal of Medical Entomology* 53:911–918. DOI:](http://paperpile.com/b/24bkyh/utfS) [10.1093/jme/tjw040.](http://dx.doi.org/10.1093/jme/tjw040.)

[Gottdenker NL., Chaves LF., Calzada JE., Saldaña A., Carroll CR. 2012. Host life history strategy, species diversity, and habitat influence *Trypanosoma cruzi* vector infection in Changing landscapes. *PLoS Neglected Tropical Diseases* 6:e1884. DOI:](http://paperpile.com/b/24bkyh/Mb6E) [10.1371/journal.pntd.0001884.](http://dx.doi.org/10.1371/journal.pntd.0001884.)

[Grijalva MJ., Palomeque FS., Villacís AG., Black CL., Arcos-Terán L. 2010. Absence of domestic triatomine colonies in an area of the coastal region of Ecuador where Chagas disease is endemic. *Memórias do Instituto Oswaldo Cruz* 105:677–681. DOI:](http://paperpile.com/b/24bkyh/Wrxh) [10.1590/S0074-02762010000500013.](http://dx.doi.org/10.1590/S0074-02762010000500013.)

[Grijalva MJ., Suarez-Davalos V., Villacis AG., Ocaña-Mayorga S., Dangles O. 2012. Ecological factors related to the widespread distribution of sylvatic *Rhodnius ecuadoriensis* populations in southern Ecuador. *Parasites & vectors* 5:17. DOI:](http://paperpile.com/b/24bkyh/rTXG) [10.1186/1756-3305-5-17.](http://dx.doi.org/10.1186/1756-3305-5-17.)

[Grijalva MJ., Villacis AG. 2009. Presence of *Rhodnius ecuadoriensis* in sylvatic habitats in the southern highlands (Loja Province) of Ecuador. *Journal of Medical Entomology* 46:708–711. DOI:](http://paperpile.com/b/24bkyh/RqPs) [10.1603/033.046.0339.](http://dx.doi.org/10.1603/033.046.0339.)

[Guarneri AA., Pereira MH., Diotaiuti L. 2000. Influence of the blood meal source on the development of *Triatoma infestans*, *Triatoma brasiliensis*, *Triatoma sordida*, and *Triatoma pseudomaculata* (Heteroptera, Reduviidae). *Journal of Medical Entomology* 37:373–379. DOI:](http://paperpile.com/b/24bkyh/rTbj) [10.1093/jmedent/37.3.373.](http://dx.doi.org/10.1093/jmedent/37.3.373.)

[Guerrero de Moyeja L., Scorza JV. 1981. Las fuentes alimenticias de algunos Triatominae silvestres en los llanos centro-occidentales de Venezuela. *Boletin de la Direccion Malariologia y Saneamiento Ambiental* 21:129–139.](http://paperpile.com/b/24bkyh/IWfJ)

[Guerrero L., García MG., Quesada MD. 1965. Campaña contra la enfermedad de Chagas. *Kasmera* 2:47–97.](http://paperpile.com/b/24bkyh/Zj3L)

[Guevara Á., Moreira J., Criollo H., Vivero S., Racines M., Cevallos V., Prandi R., Caicedo C., Robinzon F., Anselmi M. 2014. First description of *Trypanosoma cruzi* human infection in Esmeraldas province, Ecuador. *Parasites & vectors* 7:358. DOI:](http://paperpile.com/b/24bkyh/hUS8) [10.1186/1756-3305-7-358.](http://dx.doi.org/10.1186/1756-3305-7-358.)

[Guimaraes FN., Jansen G. 1943. A new wild vector of *T. cruzi* . *Memórias do Instituto Oswaldo Cruz* 38:437–441 pp.](http://paperpile.com/b/24bkyh/xhlY)

[Gurgel-Gonçalves R., Cuba CAC. 2007. Population structure of *Rhodnius neglectus* Lent and *Psammolestes tertius* Lent & Jurberg (Hemiptera, Reduviidae) in bird nests (Furnariidae) on *Mauritia flexuosa* palm trees in Federal District of Brazil. *Revista Brasileira de Zoologia* 24:157–163. DOI:](http://paperpile.com/b/24bkyh/wYea) [10.1590/S0101-81752007000100019.](http://dx.doi.org/10.1590/S0101-81752007000100019.)

[Gurgel-Gonçalves R., Cuba CAC. 2011. Infestation of thornbird nests (Passeriformes: Furnariidae) by *Psammolestes tertius* (Hemiptera: Reduviidae) across Brazilian Cerrado and Caatinga ecoregions. *Zoologia*  28:411–414. DOI:](http://paperpile.com/b/24bkyh/aOXE) [10.1590/S1984-46702011000300017.](http://dx.doi.org/10.1590/S1984-46702011000300017.)

[Gurgel-Gonçalves R., Duarte MA., Ramalho ED., Palma ART., Romaña CA., Cuba-Cuba CA. 2004. Distribuição espacial de populações de triatomíneos (Hemiptera: Reduviidae) em palmeiras da espécie *Mauritia flexuosa* no Distrito Federal, Brasil. *Revista da Sociedade Brasileira de Medicina Tropical* 37:241–247.](http://paperpile.com/b/24bkyh/vpTu)

[Gürtler RE., Cecere MC., Vazquez DP., Chuit R., Cohen JE. 1996. Host-Feeding Patterns of Domiciliary *Triatoma infestans* (Hemiptera: Reduviidae) in Northwest Argentina: Seasonal and Instar Variation. *Journal of Medical Entomology* 33:15–26. DOI:](http://paperpile.com/b/24bkyh/gEmi) [10.1093/jmedent/33.1.15.](http://dx.doi.org/10.1093/jmedent/33.1.15.)

[Gurtler RE., Cohen JE., Cecere MC., Chuit R. 1997. Shifting Host Choices of the Vector of Chagas Disease, *Triatoma Infestans*, in Relation to the Availability of Host in Houses in North-West Argentina. *The Journal of Applied Ecology* 34:699–715. DOI:](http://paperpile.com/b/24bkyh/R5zd) [10.2307/2404917.](http://dx.doi.org/10.2307/2404917.)

[Hernández C., Salazar C., Brochero H., Teherán A., Buitrago LS., Vera M., Soto H., Florez-Rivadeneira Z., Ardila S., Parra-Henao G., Ramírez JD. 2016. Untangling the transmission dynamics of primary and secondary vectors of *Trypanosoma cruzi* in Colombia: parasite infection, feeding sources and discrete typing units. *Parasites & vectors* 9:620. DOI:](http://paperpile.com/b/24bkyh/bUnz) [10.1186/s13071-016-1907-5.](http://dx.doi.org/10.1186/s13071-016-1907-5.)

[Herrer A. 1960. Geographical distribution of Chagas’ disease and of its vectors in Peru. *Boletin de la Oficina Sanitaria Panamericana. Pan American Sanitary Bureau* 49:572–581.](http://paperpile.com/b/24bkyh/0LNI)

[Herrer A., Lent H., Wygodzinsky P. 1954. Contribución al conocimiento del género *Belminus* Stal, 1859 (Triatominae, Reduviidae, Hemiptera). *An Inst Med Reg Univ Tucuman*:85–106.](http://paperpile.com/b/24bkyh/WbJL)

[Hicks EA. 1962. *Check-list and bibliography on the occurrence of insects in birds’ nest. Supplement I*. Iowa City: The Iowa State University Press.](http://paperpile.com/b/24bkyh/ku4e)

[Johnson CM., Rivas CT. 1936. La enfermedad de Chagas en Panamá. *IX Reunión Soc. Argent. Pat. Reg* 1:245.](http://paperpile.com/b/24bkyh/8WRi)

[Kjos SA., Marcet PL., Yabsley MJ., Kitron U., Snowden KF., Logan KS., Barnes JC., Dotson EM. 2013. Identification of bloodmeal sources and *Trypanosoma cruzi*  infection in triatomine bugs (Hemiptera: Reduviidae) from residential settings in Texas, the United States. *Journal of Medical Entomology* 50:1126–1139. DOI:](http://paperpile.com/b/24bkyh/6IES) [10.1603/ME12242.](http://dx.doi.org/10.1603/ME12242.)

[Klotz SA., Dorn PL., Mosbacher M., Schmidt JO. 2014. Kissing bugs in the United States: risk for vector-borne disease in humans. *Environmental Health Insights* 8:49–59. DOI:](http://paperpile.com/b/24bkyh/YnZ9) [10.4137/EHI.S16003.](http://dx.doi.org/10.4137/EHI.S16003.)

[Knierim F., Castro M., Villarroel F., Schenone H. 1976. Estudio preliminar sobre la fuente de alimentación de *Triatoma infestans* y *Triatoma spinolai* mediante la reacción de doble difusión en gel. *Boletín Chileno de Parasitología* 31:34–36.](http://paperpile.com/b/24bkyh/pZlj)

[Lent H. 1935. On the biology, classification and geographical distribution of *Psammolestes coreodes* Bergroth, 1911, found in birds’ nests in Brazil (Hem. Triatomidae). *Revista de Entomologia* 5:381–396.](http://paperpile.com/b/24bkyh/rzZx)

[Lent H. 1948. *Triatoma* and Chagas’ disease. *Proceedings 4th Intern Congr Trop Med Malaria Washington* 2:1690–1701.](http://paperpile.com/b/24bkyh/nrkl)

[Lent H., Martins AV. 1940. Estudos sobre os triatomideos do Estado de Minas Gerais, com descrição de uma espécie nova. *Rev Entomol* 11:877–886.](http://paperpile.com/b/24bkyh/Wpcs)

[Lent H., Wygodzinsky P. 1979. Revision of the Triatominae (Hemiptera, Reduviidae), and their significance as vectors of Chagas’ disease. *Bulletin of the American Museum of Natural History* 163:123–520.](http://paperpile.com/b/24bkyh/nz4N)

[de Lima JS., Rocha FL., Alves FM., Lorosa ES., Jansen AM., de Miranda Mourão G. 2015. Infestation of arboreal nests of coatis by triatomine species, vectors of *Trypanosoma cruzi* , in a large Neotropical wetland. *Journal of Vector Ecology: Journal of the Society for Vector Ecology* 40:379–385. DOI:](http://paperpile.com/b/24bkyh/v6TS) [10.1111/jvec.12177.](http://dx.doi.org/10.1111/jvec.12177.)

[Lorosa ES., Andrade RE de., Faria MS., Valente MVMP. 2008. Preferência alimentar de ninfas de 1˚ estádio de algumas espécies de triatomíneos do “complexo oliveirai” (Hemiptera, Reduviidae) em condições de laboratório. *Revista Brasileira de Zoociências* 10:163–170.](http://paperpile.com/b/24bkyh/76wG)

[Lorosa ES., Andrade RE de., Pujol-Luz JR., Jurberg J., Carcavallo RU. 2003. Determinação das fontes alimentares e da infecção natural do *Triatoma jurbergi* (Carcavallo, Galvão & Lent, 1998) *Triatoma vandae*  Carcavallo, Jurberg, Rocha, Galvão, Noireau & Lent, 2001 capturados no estado do Mato Grosso, Brasil. *Revista Brasileira de Zoociências* 5:243–252.](http://paperpile.com/b/24bkyh/tSJD)

[Lorosa ES., Andrade RE de., Rebello J., Vinhaes M da C. 1998a. Estudo das fontes alimentares através da reação de precipitina e grau de infectividade em *Triatoma rubrofasciata* (De Geer, 1773) coletado na Ilha de São Luís-Maranhão. *Entomologia y Vectores* 5:241–250.](http://paperpile.com/b/24bkyh/sUcy)

[Lorosa ES., Andrade RE de., Santos SM dos., Pereira CA. 1998b. Estudo da infecção natural e da fonte alimentar do *Triatoma sordida* (STAL, 1859),(Hemíptera-Reduviidae) na região norte de Minas Gerais, Brasil, através da reação de Precipitina. *Entomologia y Vectores* 5:13–22.](http://paperpile.com/b/24bkyh/HxAD)

[Lorosa ES., Andrade RE de., Santos SM., Pereira CA., Vinhaes M da C. 1999a. Estudo do comportamento alimentar de algumas espécies de triatomineos com auxilio da técnica de precipitina. *Entomologia y Vectores* 6:112–124.](http://paperpile.com/b/24bkyh/ebfW)

[Lorosa ES., Andrade RE de., Santos SM., Pereira CA., Vinhaes M da C., Jurberg J. 1999b. Estudo da infecção natural e fontes alimentares de *Triatoma costalimai*  Verano & Galvao, 1959, *Rhodnius neglectus*  Lent, 1954 e *Psammolestes tertius*  Lent & Jurberg, 1965 do estado de Goias, Brasil, atraves da técnica de precipitina. *Entomologia y Vectores* 6:405–414.](http://paperpile.com/b/24bkyh/VcYJ)

[Lorosa ES., Cahet D., Andrade RE de., Figueiredo JF., Jurberg J. 2000a. O uso da técnica de precipitina no estudo do comportamento alimentar e grau de infectividade em *Triatoma sordida* (Stal, 1859),(Hemiptera, Reduviidae), coletados no estado do Mato Grosso, Brasil. *Entomologia y Vectores* 7:227–237.](http://paperpile.com/b/24bkyh/k84N)

[Lorosa ES., Jurberg J., Almeida Souza AL., Vinhaes MC., Nunes IM. 2000b. Hemolinfa de Dictyoptera na manutencao do ciclo biologico silvestre de *Triatoma rubrovaria* (Blanchard 1843) e *Triatoma circummaculata* (Stal, 1859) (Hemiptera, Reduviidae, Triatominae). *Entomologia y Vectores* 7:287–296.](http://paperpile.com/b/24bkyh/Z81q)

[Lorosa ES., Nunes IM., Vinhaes M da C., de Andrade RE., Jurberg J. 2000c. Feeding preference of some species of Triatominae captured in Rio Grande do Sul State, Brazil, using the precipitin technique and level of infectivity. *Entomología y Vectores* 7:211–225.](http://paperpile.com/b/24bkyh/dN7a)

[Lucena DT. 1970. Estudos sobre a doença de Chagas no nordeste do Brasil. *Revista Brasileira de Malariologia e Doenças Tropicais. Publicações avulsas* 22:3–173.](http://paperpile.com/b/24bkyh/hoK3)

[Lucero DE., Ribera W., Pizarro JC., Plaza C., Gordon LW., Peña R Jr., Morrissey LA., Rizzo DM., Stevens L. 2014. Sources of blood meals of sylvatic *Triatoma guasayana* near Zurima, Bolivia, assayed with qPCR and 12S cloning. *PLoS Neglected Tropical Diseases* 8:e3365. DOI:](http://paperpile.com/b/24bkyh/Fui4) [10.1371/journal.pntd.0003365.](http://dx.doi.org/10.1371/journal.pntd.0003365.)

[Luz E., Borba AM. 1966. Triatomíneos encontrados no Paraná. Aspectos biológicos. *Na. Fac. Med. Univ. Fed. Paraná*.](http://paperpile.com/b/24bkyh/q6fi)

[Maes JM. 2002. Triatominae (Heteroptera Reduviidae) of Nicaragua. *Bulletin de la Société Royale Belge D’entomologie* 138:90–94.](http://paperpile.com/b/24bkyh/A7wy)

[Marcondes CB., Dias JCP., Guedes LA., Ferraz Filho AN., Rodrigues VLCC., Mendonça DD. 1991. Estudo epidemiológico de fontes de alimentação sangüínea dos triatomíneos da fazenda aroeira (Catolé do Rocha, Paraíba) e circunvizinhanças. *Revista da Sociedade Brasileira de Medicina Tropical* 24:137–140. DOI:](http://paperpile.com/b/24bkyh/6thp) [10.1590/S0037-86821991000300002.](http://dx.doi.org/10.1590/S0037-86821991000300002.)

[Marsden PD., Alvarenga NJ., Cuba CC., Shelley AJ., Costa CH., Boreham PF. 1979. Studies of the domestic ecology of *Triatoma infestans* by means of house demolition. *Revista do Instituto de Medicina Tropical de Sao Paulo* 21:13–25.](http://paperpile.com/b/24bkyh/6Vdn)

[Marti GA., Echeverria MG., Waleckx E., Susevich ML., Balsalobre A., Gorla DE. 2014. Triatominae in furnariid nests of the Argentine Gran Chaco. *Journal of Vector Ecology: Journal of the Society for Vector Ecology* 39:66–71. DOI:](http://paperpile.com/b/24bkyh/SNUj) [10.1111/j.1948-7134.2014.12071.x.](http://dx.doi.org/10.1111/j.1948-7134.2014.12071.x.)

[Martinez-Ibarra JA., Galavíz-Silva L., Campos CL., Trujillo-García JC. 1992. Distribución de los triatominos asociados al domicilio humano en el municipio de General Terán, Nuevo León, México. *The Southwestern Entomologist* 17:261–261.](http://paperpile.com/b/24bkyh/jT5X)

[Martins LPA., Castanho REP., Casanova C., Caravelas DT., Frias GT., Ruas-Neto AL., Rosa JA da. 2006. Rupestrian triatomines infected by Trypanosomatidae, collected in Quaraí, Rio Grande do Sul, 2003. *Revista da Sociedade Brasileira de Medicina Tropical* 39:198–202. DOI:](http://paperpile.com/b/24bkyh/KcbF) [10.1590/S0037-86822006000200013.](http://dx.doi.org/10.1590/S0037-86822006000200013.)

[Mascarenhas BM. 1991. Triatomíneos da Amazônia: sobre o habitat e algumas considerações comportamentais de *Rhodnius brethesi* Matta, 1919 (Hemiptera: Reduviidae: Triatominae) na região do médio Rio Negro, Amazonas. *Boletim Do Museu Paraense Emilio Goeldi Serie Zoologia* 7:107–116.](http://paperpile.com/b/24bkyh/6igP)

[Mayer HF., Alcaraz IL. 1955. Estudios relacionados con las fuentes alimentarias de *Triatoma infestans* (Hemiptera, Reduviidae). *An Inst Med Reg Tucumán* 4:195–201.](http://paperpile.com/b/24bkyh/NQeH)

[Mazza S. 1942. Remarks on Chagas’ disease in Bolivia. *Prensa Medica Argentina* 29:1–15.](http://paperpile.com/b/24bkyh/6uWC)

[Mazzotti L. 1940. Triatomideos de México y su infección natural por *Trypanosoma cruzi*, Chagas. *Medicina* 20.](http://paperpile.com/b/24bkyh/2AMw)

[Mello DA. 1981. Aspectos do ciclo silvestre do *Trypanosoma cruzi* em regiões de cerrado (Município de Formosa, Estado de Goiás). *Memórias do Instituto Oswaldo Cruz* 76:227–246. DOI:](http://paperpile.com/b/24bkyh/1SAQ) [10.1590/S0074-02761981000300001.](http://dx.doi.org/10.1590/S0074-02761981000300001.)

[Meneguetti DU de O., Tojal SD., Miranda PRM de., Rosa JA da., Camargo LMA. 2015. First report of *Rhodnius montenegrensis* (Hemiptera, Reduviidae, Triatominae) in the State of Acre, Brazil. *Revista da Sociedade Brasileira de Medicina Tropical* 48:471–473. DOI:](http://paperpile.com/b/24bkyh/zWFo) [10.1590/0037-8682-0029-2015.](http://dx.doi.org/10.1590/0037-8682-0029-2015.)

[Miles MA., Arias JR., Souza AA de. 1983. Chagas’ disease in the Amazon Basin: V. Periurban palms as habitats of *Rhodnius robustus* and *Rhodnius pictipes* - Triatomine vectors of Chagas’ disease. *Memórias do Instituto Oswaldo Cruz* 78:391–398. DOI:](http://paperpile.com/b/24bkyh/DgOl) [10.1590/S0074-02761983000400002.](http://dx.doi.org/10.1590/S0074-02761983000400002.)

[Miles MA., de Souza AA., Póvoa M. 1981. Chagas’ disease in the Amazon basin III. Ecotopes of ten triatomine bug species (Hemiptera: Reduviidae) from the vicinity of Belém, Pará State, Brazil. *Journal of Medical Entomology* 18:266–278. DOI:](http://paperpile.com/b/24bkyh/2hZm) [10.1093/jmedent/18.4.266.](http://dx.doi.org/10.1093/jmedent/18.4.266.)

[Miles MA., de Souza A., Povoa MM. 1982. The ecotope of *Panstrongylus megistus* (Hemiptera, Reduviidae) in the Horto Forest (Rio de Janeiro). *Revista Brasileira de Biologia* 42:31–36.](http://paperpile.com/b/24bkyh/ROje)

[Minter DM. 1976. Feeding patterns of some triatomine vector species. In: *New Approaches in American Trypanosomiasis Research*. Washington, D.C., Pan American Health Organization, 33–47.](http://paperpile.com/b/24bkyh/U9fL)

[Monteon V., Alducin C., Hernández J., Ramos-Ligonio A., Lopez R. 2013. High frequency of human blood in *Triatoma dimidiata* captured inside dwellings in a rural community in the Yucatan Peninsula, Mexico, but low antibody seroprevalence and electrocardiographic findings compatible with Chagas disease in humans. *The American Journal of Tropical Medicine and Hygiene* 88:566–571. DOI:](http://paperpile.com/b/24bkyh/U9Qu) [10.4269/ajtmh.12-0583.](http://dx.doi.org/10.4269/ajtmh.12-0583.)

[Monte GLS., Tadei WP., Farias TM. 2014. Ecoepidemiology and biology of *Eratyrus mucronatus* Stål, 1859 (Hemiptera: Reduviidae: Triatominae), a sylvatic vector of Chagas disease in the Brazilian Amazon. *Revista da Sociedade Brasileira de Medicina Tropical* 47:723–727. DOI:](http://paperpile.com/b/24bkyh/lLah) [10.1590/0037-8682-0263-2014.](http://dx.doi.org/10.1590/0037-8682-0263-2014.)

[Mota J., Chacón JC., Gutiérrez-Cabrera AE., Sánchez-Cordero V., Wirtz RA., Ordoñez R., Panzera F., Ramsey JM. 2007. Identification of blood meal source and infection with *Trypanosoma cruzi* of Chagas disease vectors using a multiplex cytochrome b polymerase chain reaction assay. *Vector Borne and Zoonotic Diseases*  7:617–627. DOI:](http://paperpile.com/b/24bkyh/hyW9) [10.1089/vbz.2007.0106.](http://dx.doi.org/10.1089/vbz.2007.0106.)

[Neiva A. 1914. *Revisao do genero Triatoma Lap.* Ph. D Thesis, Rio de Janeiro, Brazil.](http://paperpile.com/b/24bkyh/CoTl)

[Neiva A., Pinto C. 1923. Estado actual dos conhecimentos sôbre o gênero *Rhodnius* Stål, com a descripção de uma nova espécie. *Brasil-Médico* 37:20–24.](http://paperpile.com/b/24bkyh/h49g)

[Neiva A., Pinto C., Lent H. 1939. Notas sobre triatomideos do Rio Grande do SuI e descrição de uma nova espécie. *Memórias do Instituto Oswaldo Cruz* 34:607–610.](http://paperpile.com/b/24bkyh/mdZS)

[Noireau F., Flores R., Gutierrez T., Abad-Franch F., Flores E., Vargas F. 2000. Natural ecotopes of *Triatoma infestans* dark morph and other sylvatic triatomines in the Bolivian Chaco. *Transactions of the Royal Society of Tropical Medicine and Hygiene* 94:23–27.](http://paperpile.com/b/24bkyh/GreJ)

[Oliveira J., Alevi KCC., Fonseca EOL., Souza OMF., Santos CGS., Azeredo-Oliveira MTV., da Rosa JA. 2016. New record and cytogenetic analysis of *Psammolestes tertius* Lent & Jurberg, 1965 (Hemiptera, Reduviidae, Triatominae) from Bahia State, Brazil. *Genetics and Molecular Research: GMR* 15. DOI:](http://paperpile.com/b/24bkyh/qKfv) [10.4238/gmr.15028004.](http://dx.doi.org/10.4238/gmr.15028004.)

[Oliveira MA., Ferreira RL., Carneiro MA., Diotaiuti L. 2008. Ecology of *Cavernicola pilosa* Barber, 1937 (Hemiptera: Reduviidae: Triatominae) in the Boa Esperança Cave, Tocantins, Brazil. *Ecotropica* 14:63–68.](http://paperpile.com/b/24bkyh/sBxr)

[Packchanian A. 1939. Natural Infection of *Triatoma gerstakeri* with *Trypanosoma cruzi* in Texas. *Public Health Reports* 54:1547–1554. DOI:](http://paperpile.com/b/24bkyh/7E3h) [10.2307/4582999.](http://dx.doi.org/10.2307/4582999.)

[Patterson JS. 2007. Comparative morphometric and molecular genetic analyses of triatominae (Hemiptera : Reduviidae). Doctoral Thesis. London School of Hygiene & Tropical Medicine. DOI:](http://paperpile.com/b/24bkyh/TchR) [10.17037/PUBS.00682370 Available at https://researchonline.lshtm.ac.uk/682370/1/497303.pdf (accessed 9 August 2017).](http://dx.doi.org/10.17037/PUBS.00682370%20Available%20at%20https://researchonline.lshtm.ac.uk/682370/1/497303.pdf%20(accessed%209%20August%202017).)

[Pellegrino J. 1951. Vectors of Chagas’ disease in the state of Minas Gérais. *Revista da Associação Médica de Minas Gerais* 2:43–66.](http://paperpile.com/b/24bkyh/WKSu)

[Peña VH., Fernández GJ., Gómez-Palacio AM., Mejía-Jaramillo AM., Cantillo O., Triana-Chávez O. 2012. High-resolution melting (HRM) of the cytochrome B gene: a powerful approach to identify blood-meal sources in Chagas disease Vectors. *PLoS Neglected Tropical Diseases* 6:e1530. DOI:](http://paperpile.com/b/24bkyh/Fc35) [10.1371/journal.pntd.0001530.](http://dx.doi.org/10.1371/journal.pntd.0001530.)

[Piesman J., Sherlock IA., Christensen HA. 1983. Host availability limits population density of *Panstrongylus megistus*. *The American Journal of Tropical Medicine and Hygiene* 32:1445–1450.](http://paperpile.com/b/24bkyh/qvaT)

[Pifano FC. 1973. La dinámica epidemiológica de la enfermedad de Chagas en el Valle de Los Naranjos, Estado Carabobo, Venezuela. I. Contribución al estudio de los focos naturales silvestres del *Schizotrypanum cruzi* Chagas 1909. *Archivos Venezolanos de Medicina Tropical y Parasitologia Medica* 5:1–29.](http://paperpile.com/b/24bkyh/dBPs)

[Pineda V., Montalvo E., Alvarez D., Santamaría AM., Calzada JE., Saldaña A. 2008. Feeding sources and trypanosome infection index of *Rhodnius pallescens* in a Chagas disease endemic area of Amador County, Panama. *Revista do Instituto de Medicina Tropical de Sao Paulo* 50:113–116.](http://paperpile.com/b/24bkyh/sLWa)

[Pinto J., Cáceres AG., Vega S., Martínez R., Náquira C. 2008. Fuentes de alimentación de Panstrongylus herreri (Hemiptera: Triatominae) capturados en Utcubamba, Amazonas - Perú. *Revista Peruana de Medicina Experimental y Salud Publica* 25. DOI:](http://paperpile.com/b/24bkyh/i887) [10.17843/rpmesp.2008.252.1255.](http://dx.doi.org/10.17843/rpmesp.2008.252.1255.)

[Pinto CM., Ocaña-Mayorga S., Tapia EE., Lobos SE., Zurita AP., Aguirre-Villacís F., MacDonald A., Villacís AG., Lima L., Teixeira MMG., Grijalva MJ., Perkins SL. 2015. Bats, trypanosomes, and triatomines in Ecuador: New insights into the diversity, transmission, and origins of *Trypanosoma cruzi* and Chagas disease. *PloS One* 10:e0139999. DOI:](http://paperpile.com/b/24bkyh/0B9x) [10.1371/journal.pone.0139999.](http://dx.doi.org/10.1371/journal.pone.0139999.)

[Pipkin AC. 1962. Reduviid bugs from central Panama and incidence of infection with hemoflagellates. *The Journal of Parasitology* 48:103–111.](http://paperpile.com/b/24bkyh/xXi5)

[Pires HHR., Borges RC., de Andrade RE., Lorosa ES., Diotaiuti L. 1999. Peridomiciliary Infestation with *Triatoma sordida* Stal, 1859 in the County of Serra do Ramalho, Bahia, Brazil. *Mem Inst Oswaldo Cruz, Rio de Janeiro, Vol* 94:147–149.](http://paperpile.com/b/24bkyh/Zju8)

[Pizarro JC., Stevens L. 2008. A new method for forensic DNA analysis of the blood meal in Chagas disease vectors demonstrated using Triatoma infestans from Chuquisaca, Bolivia. *PloS One* 3:e3585. DOI:](http://paperpile.com/b/24bkyh/YOzb) [10.1371/journal.pone.0003585.](http://dx.doi.org/10.1371/journal.pone.0003585.)

[Ponce C., Trochez H., Zeledón R. 1974. Observaciones sobre enfermedad de Chagas y Tripanosomiasis rangeli en tres ranchos del Departamento Francisco Morazán, Honduras. *Revista de Biología Tropical* 22:289–301. DOI:](http://paperpile.com/b/24bkyh/zQQP) [[Chagas’ disease and trypanosomiasis rangeli in 3 ranchs of the Departmento Francisco Morazán, Honduras].](http://dx.doi.org/%5BChagas%E2%80%99%20disease%20and%20trypanosomiasis%20rangeli%20in%203%20ranchs%20of%20the%20Departmento%20Francisco%20Moraz%C3%A1n,%20Honduras%5D.)

[Quintal RE., Polanco GG. 1977. Feeding preferences of *Triatoma dimidiata maculipennis* in Yucatan, Mexico. *The American Journal of Tropical Medicine and Hygiene* 26:176–178.](http://paperpile.com/b/24bkyh/NRoe)

[Ramsey JM., Gutiérrez-Cabrera AE., Salgado-Ramírez L., Peterson AT., Sánchez-Cordero V., Ibarra-Cerdeña CN. 2012. Ecological connectivity of *Trypanosoma cruzi* reservoirs and *Triatoma pallidipennis* hosts in an anthropogenic landscape with endemic Chagas disease. *PloS One* 7:e46013. DOI:](http://paperpile.com/b/24bkyh/IR7c) [10.1371/journal.pone.0046013.](http://dx.doi.org/10.1371/journal.pone.0046013.)

[Rebêlo JMM., Barros VLL de., Mendes WA. 1998. Triatominae species (Hemiptera: Reduviidae) in Maranhão State, Brazil. *Cadernos de Saúde Pública* 14:187–192. DOI:](http://paperpile.com/b/24bkyh/00lL) [10.1590/S0102-311X1998000100027.](http://dx.doi.org/10.1590/S0102-311X1998000100027.)

[Reyes-Lugo M., Reyes-Contreras M., Salvi I., Gelves W., Avilán A., Llavaneras D., Navarrete LF., Cordero G., Sánchez EE., Rodríguez-Acosta A. 2011. The association of *Triatoma maculata* (Ericsson 1848) with the gecko *Thecadactylus rapicauda* (Houttuyn 1782) (Reptilia: Squamata: Gekkonidae): a strategy of domiciliation of the Chagas disease peridomestic vector in Venezuela? *Asian Pacific Journal of Tropical Biomedicine* 1:279–284. DOI:](http://paperpile.com/b/24bkyh/z2i2) [10.1016/S2221-1691(11)60043-9.](http://dx.doi.org/10.1016/S2221-1691(11)60043-9.)

[Reyes M., Torres Á., Esteban L., Flórez M., Angulo VM. 2017. Risk of transmission of Chagas disease by intrusion of triatomines and wild mammals in Bucaramanga, Santander, Colombia. *Biomedica: revista del Instituto Nacional de Salud* 37:68–78. DOI:](http://paperpile.com/b/24bkyh/vHh9) [10.7705/biomedica.v37i1.3051.](http://dx.doi.org/10.7705/biomedica.v37i1.3051.)

[Ribeiro G Jr., Gurgel-Gonçalves R., Reis RB., Santos CGSD., Amorim A., Andrade SG., Reis MG. 2015. Frequent house invasion of *Trypanosoma cruz*i-infected triatomines in a suburban area of Brazil. *PLoS Neglected Tropical Diseases* 9:e0003678. DOI:](http://paperpile.com/b/24bkyh/pRPv) [10.1371/journal.pntd.0003678.](http://dx.doi.org/10.1371/journal.pntd.0003678.)

[Ribeiro G Jr., Silva-Santos CG., Noireau F., Dias-Lima A. 2006. Potencial de dispersão de algumas espécies de Triatomíneos (Hemiptera: Reduviidae) por aves migratórias. *Sitientibus série Ciências Biológicas* 6:324–328.](http://paperpile.com/b/24bkyh/z7I9)

[Ricardo-Silva A., Gonçalves TCM., Luitgards-Moura JF., Lopes CM., Silva SP da., Bastos AQ., Vargas NC., Freitas M-RG. 2016. *Triatoma maculata* colonises urban domicilies in Boa Vista, Roraima, Brazil. *Memórias do Instituto Oswaldo Cruz* 111:703–706. DOI:](http://paperpile.com/b/24bkyh/imUI) [10.1590/0074-02760160026.](http://dx.doi.org/10.1590/0074-02760160026.)

[Rocha e Silva EO., Andrade JCR de., Lima AR de. 1975. Importância dos animais sinantrópicos no controle da endemia chagásica. *Revista de Saúde Pública* 9:371–381. DOI:](http://paperpile.com/b/24bkyh/hIbI) [10.1590/S0034-89101975000300010.](http://dx.doi.org/10.1590/S0034-89101975000300010.)

[Rocha e Silva EO., Souza JM., Andrade JC., Mello CS., Ferreira OA. 1977. Preferência alimentar (entre sangue humano e ave) dos *Triatoma sordida* encontrados em casas habitadas da região norte do Estado de Sao Paulo, Brasil. *Revista de Saúde Pública* 11:258–269.](http://paperpile.com/b/24bkyh/EVgR)

[Rodrigues VL., Ferraz Filho A do N., da Rocha e Silva EO., de Lima VL. 1992. The prevalence, infection indices and feeding habits of the Triatominae captured in an area of epidemiological surveillance. *Revista da Sociedade Brasileira de Medicina Tropical* 25:183–190.](http://paperpile.com/b/24bkyh/cuTb)

[Rodrigues B de A., Melo G de B. 1942. Contribuição ao estudo da tripanosomiase Americana. *Memórias do Instituto Oswaldo Cruz* 37:77–90. DOI:](http://paperpile.com/b/24bkyh/O63Y) [10.1590/S0074-02761942000100006.](http://dx.doi.org/10.1590/S0074-02761942000100006.)

[Roellig DM., Gomez-Puerta LA., Mead DG., Pinto J., Ancca-Juarez J., Calderon M., Bern C., Gilman RH., Cama VA., The Chagas Disease Workgroup. 2013. Hemi-nested PCR and RFLP methodologies for identifying blood meals of the Chagas disease vector, *Triatoma infestans*. *PloS One* 8:e74713. DOI:](http://paperpile.com/b/24bkyh/Uk7l) [10.1371/journal.pone.0074713.](http://dx.doi.org/10.1371/journal.pone.0074713.)

[Rojas-Cortez M., Pinazo M-J., Garcia L., Arteaga M., Uriona L., Gamboa S., Mejía C., Lozano D., Gascon J., Torrico F., Monteiro FA. 2016. *Trypanosoma cruzi*-infected *Panstrongylus geniculatus* and *Rhodnius robustus* adults invade households in the Tropics of Cochabamba region of Bolivia. *Parasites & Vectors* 9:158. DOI:](http://paperpile.com/b/24bkyh/e0gx) [10.1186/s13071-016-1445-1.](http://dx.doi.org/10.1186/s13071-016-1445-1.)

[Rojas JC., Malo EA., Espinoza-Medinilla E., Ondarza RN. 1989. Sylvatic focus of Chagas’ disease in Oaxaca, Mexico. *Annals of tropical medicine and parasitology* 83:115–120.](http://paperpile.com/b/24bkyh/CA4L)

[Romaña C., Abalos J. 1947. *Triatama delpontei* n. sp. (Hemiptera, Reduviidae). *An. Inst. Med. Regional* 2:79–98.](http://paperpile.com/b/24bkyh/3HYr)

[Rossell O., Mogollón J., Pacheco J. 1977. Presencia de *Rhodnius robustus* Larrousse, 1927 (Hemiptera, Reduviidae) en el estado Trujillo, Venezuela (Comunicación preliminar). *Malariol. Saneam. Amb* 17:230–233.](http://paperpile.com/b/24bkyh/3I32)

[Ruas-Neto AL., Corseuil E. 2002. Hábitos, distribuição geográfica e potencialidade dos triatomíneos rupestres como vetores da doença de Chagas no Rio Grande do Sul, Brasil (Hemiptera: Reduviidae: Triatominae). *Entomologia y Vectores* 9:231–249.](http://paperpile.com/b/24bkyh/fTnM)

[Ruas-Neto AL., Corseuil E., Cavalleri A. 2001. Development of rupestrian triatomines (Hemiptera: Reduviidae: Triatominae) following hemolymphagy on blaberids (Blattodea: Blaberidae) in Rio Grande do Sul State, Brazil. *Entomologia y Vectores* 8:205–216.](http://paperpile.com/b/24bkyh/USPB)

[Ruiz-Piña HA., Cruz-Reyes A. 2002. The opossum *Didelphis virginiana* as a synanthropic reservoir of *Trypanosoma cruzi* in Dzidzilché, Yucatán, México. *Memórias do Instituto Oswaldo Cruz* 97:613–620. DOI:](http://paperpile.com/b/24bkyh/4XxF) [10.1590/S0074-02762002000500003.](http://dx.doi.org/10.1590/S0074-02762002000500003.)

[Ryckman RE. 1951. Recent observations of cannibalism in Triatoma (Hemiptera: Reduviidae). *The Journal of Parasitology* 37:433–434.](http://paperpile.com/b/24bkyh/HU4d)

[Ryckman RE. 1962. *Biosystematics and hosts of the Triatoma protracta complex in North: (Hemiptera: Reduviidae; Rodentia: Cricetidae)*. University of California Press.](http://paperpile.com/b/24bkyh/wMRY)

[Ryckman RE. 1971. The genus *Paratriatoma* in western North America. *Journal of Medical Entomology* 8:87–97.](http://paperpile.com/b/24bkyh/QCEU)

[Ryckman RE. 1986. The vertebrate hosts of the Triatominae of North and Central America and the West Indies (Hemiptera: Reduviidae: Triatominae). *Bulletin of the Society of Vector Ecologists*.](http://paperpile.com/b/24bkyh/Nn0n)

[Ryckman RE., Ryckman AE. 1967a. Epizootiology Of *Trypanosoma cruzi* In Southwestern North America. X. The Biosystematics of *Dipetalogaster maximus* in Mexico (Hemiptera: Reduviidae) (Kinetoplastida: Trypanosomidae). *Journal of Medical Entomology* 4:180–188.](http://paperpile.com/b/24bkyh/ZTvh)

[Ryckman RE., Ryckman JV. 1967b. Epizootiology of *Trypanosoma cruzi* in Southwestern North America. XII. Does Gause’s rule apply to ectoparasitic Triatominae? (Hemiptera: Reduviidae) (Kinetoplastidae: Trypanosomidae) (Rodentia: Cricetidae). *Journal of Medical Entomology* 4:379–386.](http://paperpile.com/b/24bkyh/hjJ7)

[Sagua Franco H., Araya Rojas J., González Cortes J., Neira Cortes I. 2000. *Mepraia spinolai* in the Southeastern Pacific Ocean Coast (Chile) - First insular record and feeding pattern on the Pan de Azúcar Island. *Memórias do Instituto Oswaldo Cruz* 95:167–170. DOI:](http://paperpile.com/b/24bkyh/iMr8) [10.1590/S0074-02762000000200006.](http://dx.doi.org/10.1590/S0074-02762000000200006.)

[Salvatella R. 1986a. Triatomíneos del Uruguay. *Rev. Méd. Uruguay.* 2:106–113.](http://paperpile.com/b/24bkyh/4iCs)

[Salvatella R. 1986b. Aspectos do ciclo evolutivo de *Panstrongylus tupynambai* Lent, 1942 (Hemiptera Reduviidae) em laboratório. *Revista Brasileira de Malariologia e Doenças Tropicais. Publicações avulsas* 38:7–10.](http://paperpile.com/b/24bkyh/4HCt)

[Salvatella R. 1987. Distribución de *Triatoma platensis* Neiva, 1913 (Hemiptera-Triatominae) en Uruguay. *Revista de la Sociedad Uruguaya de Parasitologıa* 1:51–56.](http://paperpile.com/b/24bkyh/6wuu)

[Salvatella AR., Basmadjian Y., Rosa R., Puime A. 1993. *Triatoma delpontei* Romana & Abalos, 1947 (Hemiptera, Triatominae) in the Brazilian State of Rio Grande do Sul. *Revista do Instituto de Medicina Tropical de Sao Paulo* 35:73–76.](http://paperpile.com/b/24bkyh/G9iU)

[Salvatella AR., Rosa R., Basmadjian Y., Puime A., Guerrero J. 1992. *Triatoma rubrovaria* (Blanchard, 1843) (Hemiptera, Triatominae) y su alimentacion por “linfoclepsio” intraespecifico. *Boletin de la Sociedad Zoologica del Uruguay Segunda Epoca* 7:21–22.](http://paperpile.com/b/24bkyh/XeTi)

[Sandoval CM., Duarte R., Gutíerrez R., Rocha D da S., Angulo VM., Esteban L., Reyes M., Jurberg J., Galvão C. 2004. Feeding sources and natural infection of *Belminus herreri* (Hemiptera, Reduviidae, Triatominae) from dwellings in Cesar, Colombia. *Memórias do Instituto Oswaldo Cruz* 99:137–140. DOI:](http://paperpile.com/b/24bkyh/zcl7) [10.1590/S0074-02762004000200004.](http://dx.doi.org/10.1590/S0074-02762004000200004.)

[Sandoval CM., Ortiz N., Jaimes D., Lorosa E., Galvão C., Rodriguez O., Scorza JV., Gutiérrez R. 2010. Feeding behaviour of *Belminus ferroae* (Hemiptera: Reduviidae), a predaceous Triatominae colonizing rural houses in Norte de Santander, Colombia. *Medical and Veterinary Entomology* 24:124–131. DOI:](http://paperpile.com/b/24bkyh/K2ay) [10.1111/j.1365-2915.2010.00868.x.](http://dx.doi.org/10.1111/j.1365-2915.2010.00868.x.)

[Santos CB dos., Ferreira AL., Leite GR., Ferreira GEM., Rodrigues AAF., Falqueto A. 2005. Peridomiciliary colonies of *Triatoma vitticeps* (Stal, 1859) (Hemiptera, Reduviidae, Triatominae) infected with *Trypanosoma cruzi* in rural areas of the state of Espírito Santo, Brazil. *Memórias do Instituto Oswaldo Cruz* 100:471–473. DOI:](http://paperpile.com/b/24bkyh/M5wA) [10.1590/S0074-02762005000500004.](http://dx.doi.org/10.1590/S0074-02762005000500004.)

[Santos FM., Jansen AM., Mourão G de M., Jurberg J., Nunes AP., Herrera HM. 2015. Triatominae (Hemiptera, Reduviidae) in the Pantanal region: association with *Trypanosoma cruzi*, different habitats and vertebrate hosts. *Revista da Sociedade Brasileira de Medicina Tropical* 48:532–538. DOI:](http://paperpile.com/b/24bkyh/8DGh) [10.1590/0037-8682-0184-2015.](http://dx.doi.org/10.1590/0037-8682-0184-2015.)

[Santos JE dos Jr., Viola MG., Lorosa ES., Machado EM de M., Ruas Neto AL., Corseuil E. 2013. Evaluation of natural foci of *Panstrongylus megistus* in a forest fragment in Porto Alegre, State of Rio Grande do Sul, Brazil. *Revista da Sociedade Brasileira de Medicina Tropical* 46:575–583. DOI:](http://paperpile.com/b/24bkyh/ibCk) [10.1590/0037-8682-0149-2013.](http://dx.doi.org/10.1590/0037-8682-0149-2013.)

[Sarquis O., Carvalho-Costa FA., Oliveira LS., Duarte R., D Andrea PS., de Oliveira TG., Lima MM. 2010. Ecology of *Triatoma brasiliensis* in northeastern Brazil: seasonal distribution, feeding resources, and *Trypanosoma cruzi* infection in a sylvatic population. *Journal of Vector ecology: Journal of the Society for Vector Ecology* 35:385–394. DOI:](http://paperpile.com/b/24bkyh/AvLL) [10.1111/j.1948-7134.2010.00097.x.](http://dx.doi.org/10.1111/j.1948-7134.2010.00097.x.)

[Sarquis O., Sposina R., de Oliveira TG., Mac Cord JR., Cabello PH., Borges-Pereira J., Lima MM. 2006. Aspects of peridomiciliary ecotopes in rural areas of northeastern Brazil associated to triatomine (Hemiptera, Reduviidae) infestation, vectors of chagas disease. *Memórias do Instituto Oswaldo Cruz* 101:143–147.](http://paperpile.com/b/24bkyh/kJ55)

[Sasaki H., Rosales R., Tabaru Y. 2003. Host feeding profiles of *Rhodnius prolixus* and *Triatoma dimidiata* in Guatemala (Hemiptera: Reduviidae: Triatominae). *Medical Entomology and Zoology* 54:283–289. DOI:](http://paperpile.com/b/24bkyh/rVDZ) [10.7601/mez.54.283.](http://dx.doi.org/10.7601/mez.54.283.)

[Schenone H., Christensen H A., de Vasquez A M. 1985. Feeding sources of domestic Triatominae and its epidemiological implication in relation to Chagas’ disease in rural sections of seven regions of Chile. *Boletín Chileno de Parasitologia* 40:34–38.](http://paperpile.com/b/24bkyh/oTW0)

[Schofield CJ., Marsden PD., Das VD. 1980. Notes on the biology of *Triatoma costalimai* Verano and Galvao, 1958. (Hemiptera; Reduviidae; Triatominae). *Anais da Sociedade Entomologica do Brasil* 9:295–301.](http://paperpile.com/b/24bkyh/gtPm)

[Sherlock IA., Serafim. EM. 1967. *Triatoma lenti* sp. n. *Triatoma pessoai* sp. n. and *Triatoma bahiensis* sp. n. from the state of Bahia Brazil. *Gazeta Medica da Bahia* 67:75–92.](http://paperpile.com/b/24bkyh/G9J1)

[Sherlock IA., Serafim EM. 1972. Fauna Triatominae no Estado da Bahia, Brasil: as espécies e distribuição geográfica. *Revista da Sociedade Brasileira de Medicina Tropical* 6:265–276. DOI:](http://paperpile.com/b/24bkyh/RkRK) [10.1590/S0037-86821972000500005.](http://dx.doi.org/10.1590/S0037-86821972000500005.)

[Soares RP., Barbosa SE., Borges EC., Melo Júnior TA., Romanha AJ., Dujardin JP., Schofield CJ., Diotaiuti L. 2001. Genetic studies of *Psammolestes tertius* (Hemíptera: Reduviidae: Triatominae) using male genital morphology, morphometry, isoenzymes, and random amplified polymorphic DNA. *Biochemical Genetics* 39:1–13.](http://paperpile.com/b/24bkyh/NnDy)

[Sousa OE., Galindo P. 1972. Natural infections of *Triatoma dispar* Lent 1950 with *Trypanosoma cruzi* in Panama. *The American Journal of Tropical Medicine and Hygiene* 21:293–295.](http://paperpile.com/b/24bkyh/fbZT)

[Souza R de CM de., Soares AC., Alves CL., Lorosa ES., Pereira MH., Diotaiuti L. 2011. Feeding behavior of *Triatoma vitticeps* (Reduviidae: Triatominae) in the state of Minas Gerais, Brazil. *Memórias do Instituto Oswaldo Cruz* 106:16–22. DOI:](http://paperpile.com/b/24bkyh/NkCC) [10.1590/S0074-02762011000100003.](http://dx.doi.org/10.1590/S0074-02762011000100003.)

[Steindel M., Toma HK., Carvalho Pinto CJ de., Grisard EC., Schlemper BR Jr. 1994. Colonization of artificial ecotopes by *Panstrongylus megistus* at Santa Catarina Island, Florianópolis, Santa Catarina, Brazil. *Revista do Instituto de Medicina Tropical de Sao Paulo* 36:43–50. DOI:](http://paperpile.com/b/24bkyh/1on4) [10.1590/S0036-46651994000100008.](http://dx.doi.org/10.1590/S0036-46651994000100008.)

[Stevens L., Dorn PL., Hobson J., de la Rua NM., Lucero DE., Klotz JH., Schmidt JO., Klotz SA. 2012. Vector blood meals and Chagas disease transmission potential, United States. *Emerging Infectious Diseases* 18:646–649. DOI:](http://paperpile.com/b/24bkyh/7WsH) [10.3201/eid1804.111396.](http://dx.doi.org/10.3201/eid1804.111396.)

[Stevens L., Monroy MC., Rodas AG., Dorn PL. 2014. Hunting, swimming, and worshiping: human cultural practices illuminate the blood meal sources of cave dwelling Chagas vectors (*Triatoma dimidiata*) in Guatemala and Belize. *PLoS Neglected Tropical Diseases* 8:e3047. DOI:](http://paperpile.com/b/24bkyh/zYOG) [10.1371/journal.pntd.0003047.](http://dx.doi.org/10.1371/journal.pntd.0003047.)

[Suarez-Davalos V., Dangles O., Villacis AG., Grijalva MJ. 2010. Microdistribution of sylvatic triatomine populations in central-coastal Ecuador. *Journal of Medical Entomology* 47:80–88.](http://paperpile.com/b/24bkyh/vkf6)

[Tay J. 1981. La enfermedad de Chagas en la República Mexicana. *Salud Pública de México* 22:409–450.](http://paperpile.com/b/24bkyh/TFw4)

[Teixeira AR., Monteiro PS., Rebelo JM., Argañaraz ER., Vieira D., Lauria-Pires L., Nascimento R., Vexenat CA., Silva AR., Ault SK., Costa JM. 2001. Emerging Chagas disease: trophic network and cycle of transmission of *Trypanosoma cruzi* from palm trees in the Amazon. *Emerging Infectious Diseases* 7:100–112. DOI:](http://paperpile.com/b/24bkyh/7aZg) [10.3201/eid0701.700100.](http://dx.doi.org/10.3201/eid0701.700100.)

[Tonn RJ. 1978. Aspectos biológicos, ecológicos y distribución geográfica de *Triatoma maculata* (Erichson, 1848), (Hemiptera, Reduviidae), en Venezuela. *Boletin de la Direccion de Malariologia y Saneamiento Ambiental* 18:16–24.](http://paperpile.com/b/24bkyh/5r1l)

[Tonn RJ., Carcavallo RU., Ortega R. 1976. Notes on the biology, ecology and geographical distribution of *Rhodnius robustus* (Hemiptera, Reduviidae). *Boletin de la Direccion de Malariologia y Saneamiento Ambiental* 16:158–162.](http://paperpile.com/b/24bkyh/ywpC)

[Tonn R., Espinola H., Boteham P., Mora E., Otero M. 1982. Fuentes de alimentación sanguínea de ciertos triatóminos en Venezuela. *Bol. Dir. Malariol. San. Amb* 22:45–52.](http://paperpile.com/b/24bkyh/KAeA)

[Torrealba JW. 1970. Unpublished data cited in Feeding patterns of Triatominae in relation to transmission of American trypanosomiasis. By: Wisnivesky-Colli, C Edited by: Brenner, RR; de la Merced Stoka, A 1987. Chagas’ disease vectors. Volume 1. Taxonomic, ecological and epidemiological aspects. Pages: 99-117.](http://paperpile.com/b/24bkyh/qwJZ)

[Torres-Montero J., López-Monteon A., Dumonteil E., Ramos-Ligonio A. 2012. House infestation dynamics and feeding sources of *Triatoma dimidiata* in central Veracruz, Mexico. *The American Journal of Tropical Medicine and Hygiene* 86:677–682. DOI:](http://paperpile.com/b/24bkyh/7mYc) [10.4269/ajtmh.2012.11-0746.](http://dx.doi.org/10.4269/ajtmh.2012.11-0746.)

[Turienzo P., Di Iorio O. 2014. Insects found in birds’ nests from Argentina: *Coryphistera alaudina* Burmeister, 1860 (Aves: Furnariidae), their inquiline birds and mammals, new hosts for *Psammolestes coreodes* Bergroth, 1911 and *Triatoma platensis* Neiva, 1913 (Hemiptera: Reduviidae: Triatominae). *Zootaxa*:151–184. DOI:](http://paperpile.com/b/24bkyh/VW3A) [10.11646/zootaxa.3811.2.1.](http://dx.doi.org/10.11646/zootaxa.3811.2.1.)

[Usinger RL. 1944. The triatominae of North and Central America and the West Indies and their public health significance. *Public Health Bulletin* 288:1–83.](http://paperpile.com/b/24bkyh/L1j6)

[Valença-Barbosa C., Fernandes FA., Santos HLC., Sarquis O., Harry M., Almeida CE., Lima MM. 2015. Molecular identification of food sources in Triatomines in the Brazilian northeast: Roles of goats and rodents in Chagas disease epidemiology. *The American Journal of Tropical Medicine and Hygiene* 93:994–997. DOI:](http://paperpile.com/b/24bkyh/IyMw) [10.4269/ajtmh.15-0156.](http://dx.doi.org/10.4269/ajtmh.15-0156.)

[Valença-Barbosa C., Lima MM., Sarquis O., Bezerra CM., Abad-Franch F. 2014a. Modeling disease vector occurrence when detection is imperfect II: Drivers of site-occupancy by synanthropic *Triatoma brasiliensis* in the Brazilian northeast. *PLoS Neglected Tropical Diseases* 8:e2861. DOI:](http://paperpile.com/b/24bkyh/espZ) [10.1371/journal.pntd.0002861.](http://dx.doi.org/10.1371/journal.pntd.0002861.)

[Valença-Barbosa C., Lima MM., Sarquis O., Bezerra CM., Abad-Franch F. 2014b. A common Caatinga cactus, *Pilosocereus gounellei*, is an important ecotope of wild *Triatoma brasiliensis* populations in the Jaguaribe valley of northeastern Brazil. *The American Journal of Tropical Medicine and Hygiene* 90:1059–1062. DOI:](http://paperpile.com/b/24bkyh/bkgR) [10.4269/ajtmh.13-0204.](http://dx.doi.org/10.4269/ajtmh.13-0204.)

[Vallvé SL., Rojo H., Wisnivesky-Colli C. 1995. Ecología urbana de *Triatoma infestans* en Argentina: asociación entre *Triatoma infestans* y palomares. *Revista de Saúde Pública* 29:192–198. DOI:](http://paperpile.com/b/24bkyh/lXCr) [10.1590/S0034-89101995000300006.](http://dx.doi.org/10.1590/S0034-89101995000300006.)

[Vargas MV., Montero-Gei F. 1971. *Triatoma dispar* Lent, 1950 in Costa Rica (Hemiptera, Reduviidae). *Journal of Medical Entomology* 8:454–455. DOI:](http://paperpile.com/b/24bkyh/fLNs) [10.1093/jmedent/8.4.454.](http://dx.doi.org/10.1093/jmedent/8.4.454.)

[Verano OT., Galvão AB. 1958. *Triatoma costalimai* sp., n. *Revista Brasileira de Malariologia e*](http://paperpile.com/b/24bkyh/KDh6) [*Doenças*](http://paperpile.com/b/24bkyh/4HCt) [*Tropicais. Publicacoes avulsas* 10:199–205.](http://paperpile.com/b/24bkyh/KDh6)

[Villacís AG., Ocaña-Mayorga S., Lascano MS., Yumiseva CA., Baus EG., Grijalva MJ. 2015. Abundance, natural infection with trypanosomes, and food source of an endemic species of triatomine, *Panstrongylus howardi* (Neiva 1911), on the Ecuadorian Central Coast. *The American Journal of Tropical Medicine and Hygiene* 92:187–192. DOI:](http://paperpile.com/b/24bkyh/A7c1) [10.4269/ajtmh.14-0250.](http://dx.doi.org/10.4269/ajtmh.14-0250.)

[Villela MM., Rodrigues VLCC., Casanova C., Dias JCP. 2010. Analysis on the food source of *Panstrongylus megistus* (Hemiptera, Reduviidae, Triatominae) and its present importance as a vector for *Trypanosoma cruzi*, in the State of Minas Gerais. *Revista da Sociedade Brasileira de Medicina Tropical* 43:125–128.](http://paperpile.com/b/24bkyh/2wac)

[Waleckx E., Suarez J., Richards B., Dorn PL. 2014. *Triatoma sanguisuga* blood meals and potential for Chagas disease, Louisiana, USA. *Emerging Infectious Disease journal* 20:2141. DOI:](http://paperpile.com/b/24bkyh/NfQh) [10.3201/eid2012.131576.](http://dx.doi.org/10.3201/eid2012.131576.)

[Wanderley D., Carvalho ME., Silva RA., Rodrigues VL., Barbosa L., Curado I. 2006. Programa de Controle da Doença de Chagas. *Boletim Epidemiológico Paulista* 3:13–18.](http://paperpile.com/b/24bkyh/6wkG)

[Wier-López EH. 1982. Estado alimentario y regulación poblacional en *Rhodnius prolixus* (Hemiptera: Reduviidae). PhD Thesis Thesis. Universidad Simón Bolívar, Caracas, Venezuela.](http://paperpile.com/b/24bkyh/ALDU)

[Wilson GT., Garippa D., Shake R., Manis A. 1981. Search of wood rat (*Neotoma*) nests in Taylor and Jones Counties, Texas, for the presence of Reduviidae infected with *Trypanosoma cruzi*. *The Southwestern Naturalist* 25:565–566.](http://paperpile.com/b/24bkyh/9tPs)

[Wisnivesky-Colli C., Gürtler RE., Solarz N., Salomón D., Ruiz A. 1982. Feeding patterns of *Triatoma infestans* (Hemiptera: Reduviidae) in relation to transmission of American trypanosomiasis in Argentina. *Journal of Medical Entomology* 19:645–654.](http://paperpile.com/b/24bkyh/3vW7)

[Wood SF. 1941. Notes on the distribution and habits of Reduviid vectors of Chagas’ Disease in the southwestern United States (Hemiptera, Reduviidae). *The Pan-Pacific Entomologist* 17:85–94.](http://paperpile.com/b/24bkyh/Gd2c)

[Wood SF. 1946. The occurrence of *Trypanosoma conorhini* Donovan in the Reduviid bug, *Triatoma rubrofasciata* (Degeer) from Oahu, T. H. *Proceedings of the Hawaiian Entomological Society* 12:651 p.](http://paperpile.com/b/24bkyh/GEUU)

[Wood SF., Wood FD. 1961. Observations on vectors of Chagas’ disease in the United States. III. New Mexico. *The American Journal of Tropical Medicine and Hygiene* 10:155–165.](http://paperpile.com/b/24bkyh/wl0C)

[Zarate LG., Zarate RJ. 1985. A checklist of the Triatominae (Hemiptera, reduviidae) of Mexico. *International Journal of Industrial Entomology* 27:102–127.](http://paperpile.com/b/24bkyh/ts7G)

[Zárate LG., Zárate RJ., Tempelis CH., Goldsmith RS. 1980. The biology and behavior of *Triatoma barberi* (Hemiptera: Reduviidae) in Mexico. I. Blood meal sources and infection with *Trypanosoma cruzi*. *Journal of Medical Entomology* 17:103–116.](http://paperpile.com/b/24bkyh/1rGx)

[Zavala-Velázquez J., Barrera-Pérez M., Rodríguez-Félix ME., Guzmán-Marín E., Ruíz-Piña H. 1996. Infection by *Trypanosoma* cruzi in mammals in Yucatan, Mexico: a serological and parasitological study. *Revista do Instituto de Medicina Tropical de Sao Paulo* 38:289–292.](http://paperpile.com/b/24bkyh/HCwQ)

[Zehntner MJP. 2011. Identificación de fuentes alimenticias y de la presencia de *Trypanosoma cruzi* utilizando la reacción en cadena de la polimerasa (PCR) del ADN en la ingesta de sangre de *Triatoma dimidiata* de colectas de las aldeas La Brea y el Tule del municipio de Quesada, Jutiapa, Guatemala, antes y después de modificaciones en ecotopos domiciliares y peridomiciliares. Ph. D. Thesis Thesis. Universidad de San Carlos de Guatemala. Available at http://biblioteca.usac.edu.gt/tesis/06/06_3219.pdf (accessed 9 August 2017).](http://paperpile.com/b/24bkyh/zFsQ)

[Zeledón R., Calvo N., Montenegro VM., Lorosa ES., Arévalo C. 2005. A survey on *Triatoma dimidiata* in an urban area of the province of Heredia, Costa Rica. *Memórias do Instituto Oswaldo Cruz* 100:507–512. DOI:](http://paperpile.com/b/24bkyh/hZpp) [/S0074-02762005000600002.](http://dx.doi.org//S0074-02762005000600002.)

[Zeledón R., Cordero M., Marroquín R., Lorosa ES. 2010. Life cycle of *Triatoma ryckmani* (Hemiptera: Reduviidae) in the laboratory, feeding patterns in nature and experimental infection with *Trypanosoma cruzi*. *Memórias do Instituto Oswaldo Cruz* 105:99–102.](http://paperpile.com/b/24bkyh/Geqq)

[Zeledón R., Rabinovich JE. 1981. Chagas Disease: an ecological appraisal with special emphasis on its insect vectors. *Annual Review of Entomology* 26:101–133. DOI:](http://paperpile.com/b/24bkyh/SOM8) [10.1146/annurev.en.26.010181.000533.](http://dx.doi.org/10.1146/annurev.en.26.010181.000533.)

[Zeledón R., Solano G., Swartzwelder JC. 1970. Sources of blood for *Triatoma dimidiata* (Hemiptera: Reduviidae) in an endemic area of Chagas’ disease in Costa Rica. *The Journal of Parasitology* 56:102.](http://paperpile.com/b/24bkyh/9ChG)

[Zeledón R., Solano G., Zúñiga A., Swartzwelder JC. 1973. Biology and ethology of *Triatoma dimidiata* (Latreille, 1811). 3. Habitat and blood sources. *Journal of Medical Entomology* 10:363–370. DOI:](http://paperpile.com/b/24bkyh/FY7A) [10.1093/jmedent/10.4.363.](http://dx.doi.org/10.1093/jmedent/10.4.363.)
